# Great egret (*Ardea alba*) habitat selection and foraging behavior in a temperate estuary: Comparing natural wetlands to areas with shellfish aquaculture

**DOI:** 10.1371/journal.pone.0261963

**Published:** 2021-12-31

**Authors:** Scott Jennings, David Lumpkin, Nils Warnock, T. Emiko Condeso, John P. Kelly

**Affiliations:** Cypress Grove Research Center, Audubon Canyon Ranch, Marshall, California, United States of America; Kaohsiung Medical University, TAIWAN

## Abstract

Movement by animals to obtain resources and avoid predation often depends on natural cycles, and human alteration of the landscape may disrupt or enhance the utility of different habitats or resources to animals through the phases of these cycles. We studied habitat selection by GPS/accelerometer-tagged great egrets (*Ardea alba*) foraging in areas with shellfish aquaculture infrastructure and adjacent natural wetlands, while accounting for tide-based changes in water depth. We used integrated step selection analysis to test the prediction that egrets would express stronger selection for natural wetlands (eelgrass, tidal marsh, and other tidal wetlands) than for shellfish aquaculture areas. We also evaluated differences in foraging behavior among shellfish aquaculture areas and natural wetlands by comparing speed travelled (estimated from distance between GPS locations) and energy expended (Overall Dynamic Body Acceleration) while foraging. We found evidence for stronger overall habitat selection for eelgrass than for shellfish aquaculture areas, with results conditional on water depth: egrets used shellfish aquaculture areas, but only within a much narrower range of water depths than they used eelgrass and other natural wetlands. We found only slight differences in our metrics of foraging behavior among shellfish aquaculture areas and natural wetlands. Our results suggest that although great egrets appear to perceive or experience shellfish aquaculture areas as suitable foraging habitat during some conditions, those areas provide less foraging opportunity throughout tidal cycles than natural wetlands. Thus, expanding the footprint of shellfish aquaculture into additional intertidal areas may reduce foraging opportunities for great egrets across the range of tidal cycles. Over longer time scales, the ways in which natural wetlands and shellfish aquaculture areas adapt to rising sea levels (either through passive processes or active management) may change the ratios of these wetland types and consequently change the overall value of Tomales Bay to foraging great egrets.

## Introduction

Motile organisms have the capacity to change the environmental conditions they experience through movement [1]. Often, these movements are tuned to natural cycles (e.g. seasonal, circadian, tidal) that drive resource availability or abundance (e.g. [2–4]). Human alteration of natural landscapes can alter animal movement behaviors and the ecological roles animals play. For example, human-altered landscapes were associated with reduced distance travelled across a range of mammalian taxa [5] and reduced connectivity within a population of birds [6]. In production landscapes where industry or agriculture can alter the natural availability of resources, animals may spend more time and energy travelling to find food and to avoid disturbance, and this may be especially true for larger, more mobile animals [7]. The impact of human landscape alteration on habitat and resource availability at certain times of seasonal migration has been well studied [8], but the interaction of human impacts with movement cycles spanning shorter time scales is less well understood.

Great egrets (*Ardea alba*) are mobile, generalist wetland predators found in temperate and tropical latitudes throughout the world [9]. They exist in coastal and inland areas and forage in a wide variety of freshwater, estuarine, and marine wetlands and some upland habitats. Great egrets are opportunistic foragers and will select foraging habitat where environmental or human-caused factors enhance prey abundance and availability [10]. They employ a range of foraging behaviors, most commonly slow-walking or sit-and-wait [9], and foraging behavior may be related to habitat type [11]. Formal energetic investigation has revealed that walking and striking at prey is inexpensive for great egrets relative to the energy gained from prey. As a result, energy gain is positively related to the number of strikes at prey and the number of steps taken [12]. However, it remains unclear under what circumstances different feeding activities are more profitable for great egrets and whether any differences may be influenced by habitat type.

Great egrets and other wading birds (Ardeidae) are well known to forage at finfish aquaculture facilities, and extensive research has been conducted and effort expended to reduce the economic impact of these foraging behaviors [13, 14]. Less attention has been given to the relationships between wading birds and shellfish aquaculture. Shellfish aquaculture can provide habitat for nekton [15], which may in turn serve as valuable prey for wading birds. However, if the structure of aquaculture equipment provides sufficient cover, even abundant prey may nevertheless be unavailable to wading birds [16]. Additionally, increased human activity associated with maintaining aquaculture infrastructure may dissuade wading birds from foraging there. Commercial shellfish harvest on Tomales Bay, Marin Co., CA, has increased more than four-fold since 1990 [17]. However, the degree to which these activities alter the value of intertidal areas as wildlife habitat remains understudied, limiting the empirical evidence available to agencies responsible for regulating this industry.

Extensive eelgrass (*Zostera* spp.) beds exist on Tomales Bay, often directly adjacent to shellfish aquaculture infrastructure. In coastal systems, eelgrass can provide important foraging areas for great egrets [18]. These habitats also provide important ecosystem services, including habitat for economically and culturally important species [19, 20], long-term sequestration of blue carbon [21], and buffering of nutrient pulses from terrestrial to marine systems [22]. Eelgrass habitats are threatened by a range of human activities and receive considerable attention by conservation and regulatory entities (e.g. [23]). A better understanding of the ways in which shellfish aquaculture facilities and infrastructure might operate as surrogates for natural habitat for foraging wading birds is important for determining reasonable limits on the loss or alteration of natural habitat. Additionally, because top predators, including wading birds, may exert top-down regulation of processes in natural systems like eelgrass [24, 25], it is important to understand the degree to which aquaculture operations adjacent to natural areas may alter those regulating effects.

We used GPS/Accelerometer dataloggers to quantify great egret habitat selection and foraging behavior in four types of tidal wetlands on Tomales Bay: eelgrass, tidal marsh, other (mostly unvegetated mudflat) natural tidal, and shellfish aquaculture areas. Our objectives were two-fold. First, we tested an *a priori* prediction about relative selection of tidal habitats on Tomales Bay. We hypothesized that great egrets perceive or experience eelgrass and other natural wetlands as higher quality foraging habitat than shellfish aquaculture areas. Based on this hypothesis we expected foraging great egrets to select natural wetlands, and especially eelgrass beds, more strongly than areas occupied by shellfish aquaculture infrastructure. Second, we quantitatively described egret foraging movement and behavior in these areas. The literature contains conflicting information about how behaviors (as measured with GPS/Accelerometry) might translate to true foraging success or other fitness metrics, so we did not develop and test formal predictions. Rather, our aim for this component of our study was descriptive and intended to generate hypotheses about behavioral differences by great egrets in shellfish aquaculture areas relative to natural tidal wetlands. To address this objective, we compared two quantitative measures of foraging behavior between wetland types: 1) foraging speed; and 2) energy expenditure.

## Methods

### Study area

**Fig 1:**
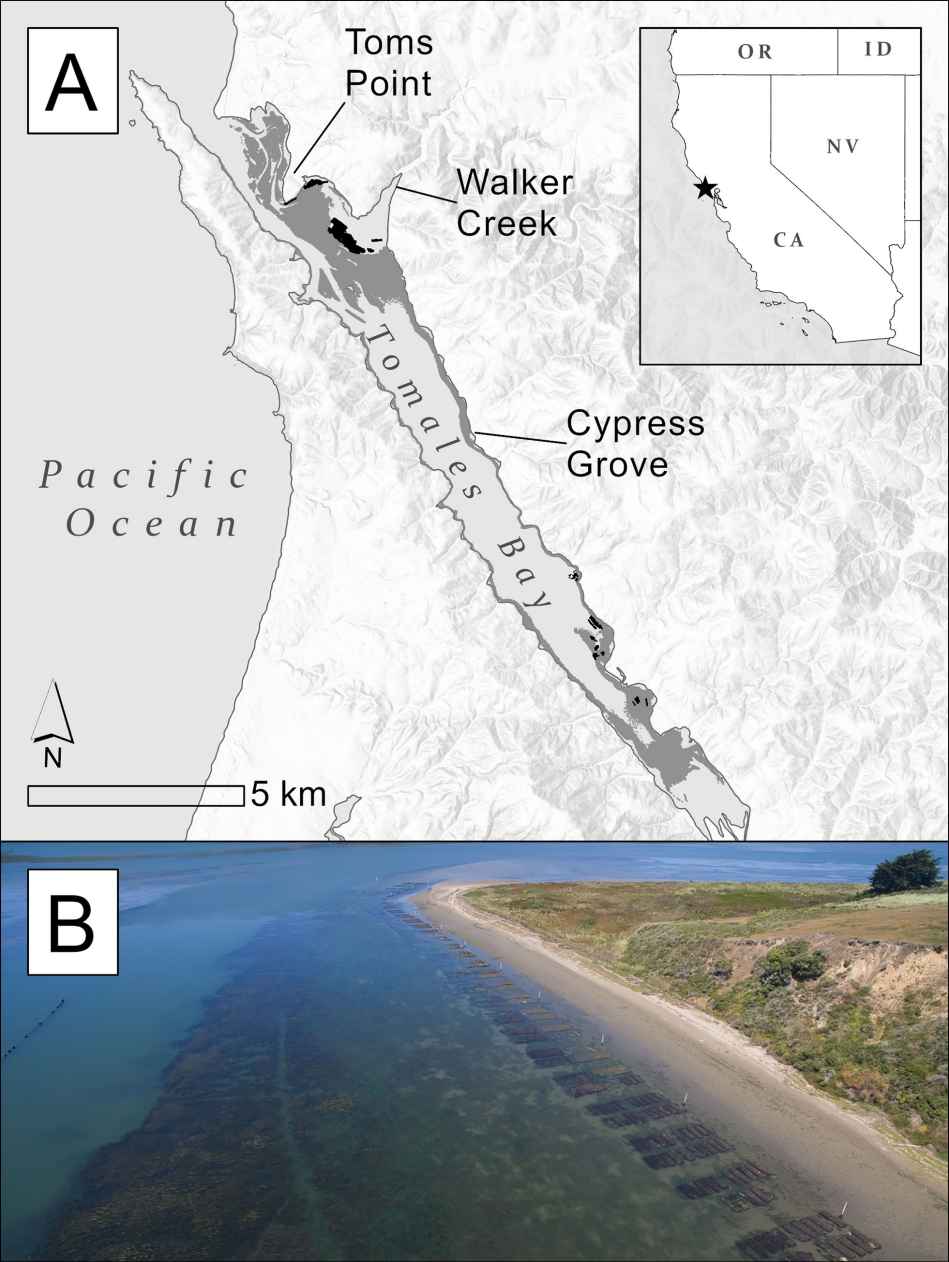
Study site map. A: Tomales Bay, CA, where selection and foraging behavior of GPS-tagged great egrets was investigated from 2017 to 2020. The three trapping locations are indicated with a line terminating at the location. Eelgrass is shown in dark gray and shellfish aquaculture infrastructure shown in black. B: Aerial photo looking southwest along the southern tip of Toms Point, showing a typical arrangement of shellfish aquaculture infrastructure and eelgrass beds (submerged vegetation bayward from shellfish gear). Copyright © ESRI. All rights reserved. Photo credit: Richard James/coastodian.org.

Tomales Bay (38.161921° N, -122.905464° W) is a linear estuary in Marin Co., CA, USA. It covers an area totaling approximately 31.9 km^2^ [26] and is permanently open to the Pacific Ocean at its northern end (Fig 1). The bay is mostly shallow (mean depth <6.5 m below Mean Lower Low Water [MLLW]), and of the total area approximately 14.7 km^2^ is shallow enough for great egret foraging at some point in the tidal range (see below). We defined these 14.7 km^2^ of tidal and subtidal wetlands as our study area. Of these 14.7 km^2^, approximately 5.3 km^2^ (35.8%) is covered by eelgrass, 0.3 km^2^ (2.3%) by shellfish aquaculture infrastructure, 3.0 km^2^ (20.9%) by tidal marsh, and the remaining 6.0 km^2^ (41.1%) by mostly unvegetated intertidal and subtidal flats (see below for wetland classification). The most extensive shellfish aquaculture infrastructure in areas shallow enough for egrets to forage is in the northern part of the bay, near Walker Creek delta and Toms Point (Fig 1).

### Trapping/Tagging

Great egrets were trapped and tagged at three locations within Tomales Bay: Toms Point, Walker Creek, and Cypress Grove (Fig 1). Trapping and banding methods conformed to the Ornithological Councils guidelines to the use of wild birds in research [27]. Great egrets were captured and tagged under the United States Department of Interior federal bird banding permit #24179 and the California Department of Fish and Wildlife’s Scientific Collecting Permit #SC-1383.

We lured egrets using decoys and bait and trapped them in padded leghold traps (Victor #3 Soft Catch Coil Spring Trap) following established methods for wading birds [28]. Traps were modified to close with less force and not close tighter than the diameter of egret legs. We removed birds from traps immediately upon capture. We attached GPS tags (Bird Solar 48g or Bird UMTS 25g, e-obs GmbH, Gruenwald, Germany) using a backpack harness of Teflon ribbon (Bally Ribbon Mills, PA, USA). We programmed the tags to collect a GPS location and a 6 sec burst (10 Hz per axis sampling rate) of acceleration data at 5 min intervals.

### Data preparation

#### Habitat mapping

Our analysis required three types of environmental data: wetland type, elevation, and predicted tide height. We combined data from the following three sources to assign wetland type to GPS locations.

*Wetlands*. We used the California Aquatic Resources Inventory dataset (CARI), a publicly available habitat classification [26] to assign habitat values to each GPS location. The original CARI dataset segregated habitat into finer categories than necessary for our analysis. We retained the “tidal marsh” classification but combined unvegetated intertidal and subtidal areas into “other tidal.” We excluded areas classified as freshwater wetlands and non-wetland areas from this analysis.

*Eelgrass*. We also acquired a GIS layer representing the areal extent of eelgrass in Tomales Bay [29]. These data were collected in 2017 by a combination of vessel-borne side-scan sonar at high tides and low altitude unmanned aerial vehicle photography at low tide. We buffered the resulting eelgrass layer by 5m to close gaps between shapes representing small adjacent eelgrass patches. This meant more of these small patches were included in the eelgrass layer when converted to the 10 m^2^ raster (see below), but it also meant that we classified more non-eelgrass areas as eelgrass than vice versa.

*Shellfish aquaculture infrastructure*. We digitized all visually apparent structures associated with shellfish aquaculture on the bay using 15.25 cm resolution ortho images [30] viewed in a GIS at scales between 1:300 and 1:3000. Shellfish aquaculture on Tomales Bay primarily uses tipping baskets, which are plastic mesh envelopes approximately 40 by 80 cm, either strung together in lines and allowed to rest on the bottom or suspended off the bottom by risers. We digitized visible aquaculture infrastructure using a combination of polygon and linear features: long lines of baskets (generally appearing <1m wide) separated by >2m were represented by lines then enclosed in a 1m buffer (resulting in a polygon). Where there was <2m between visible gear we enclosed the visible gear in shared polygons. Until just prior to our study, clams were cultured in cylindrical bags that become buried in the sediment; we also used polygons to enclose areas where substrate scars remaining from this activity were visible in the aerial images. All polygons were then merged, and we buffered the resulting shape by 1m to close small interior gaps.

*Elevation*. Although our study area extended above sea level, where height is termed “topographic elevation”, and below sea level, where height is termed “bathymetric depth”, we use “elevation” to represent both. We used two data sources for elevation. Our primary elevation source was a LiDAR-derived digital elevation map (DEM) [31]. However, this map did not provide accurate elevation below Mean Lower Low Water (MLLW). Thus, for areas below that elevation we used a Tomales Bay bathymetry layer [32].

*Tide height*. There is no tide gauge on Tomales Bay. We generated predicted tide height by offsetting hourly predictions from the San Francisco tide station (NOAA station #9414290) by the published time and height values for the mid-bay subordinate station at Blake’s Landing (station #9415396). From these hourly predictions we then interpolated tide estimates for the timestamp associated with each GPS location collected by the tags.

*Combining layers*. Finally, the CARI, eelgrass and shellfish layers were converted to rasters with 10m cell size, then combined such that CARI areas were reclassified as either eelgrass or shellfish where the later datasets existed; where the eelgrass and shellfish rasters contained values of NA, the CARI values were retained. Thus, our combined layer classified tidal wetlands into four categories: eelgrass, shellfish, tidal marsh (any vegetated tidal area not contained in the eelgrass layer), and other tidal (almost entirely unvegetated inter- and subtidal mud flats). In all analyses, other tidal was coded as the reference level for the wetland type variable.

We calculated water depth as the predicted tide height at the timestamp associated with each GPS fix minus the elevation at that GPS fix. The 0 values for the datum for the DEM (NAVD88 GEOID 12B) and that for the bathymetric and tide predictions (MLLW) differed by < 1 cm, a difference we deemed small enough to not warrant converting to make both equal. Our calculated water depth was specific to the location and time that each GPS fix was collected. The same location would experience different water depths through the tide cycle, and an individual egret could experience different water depths at a particular time by moving up or down in elevation. Furthermore, our water depth variable contained negative values, which represented the height above the water level at each specific GPS location and timestamp. Thus, a given location at 0.5 m elevation would have a water depth of 0.3 m at a tide height of 0.8 m, a water depth of 0 m at a tide height of 0.5 m, and a water depth of -0.5 m at a tide height of 0 m.

#### GPS/Accelerometer data

We first filtered GPS location data to just those points with tag-estimated accuracy < 10m (best accuracy was ~3m). Because our focus was on foraging habitat selection, we further filtered GPS data to include only locations collected during daylight hours (when great egrets forage) and when the bird’s speed (as estimated by the GPS unit) was ≤ 5 m s^-1^ to exclude locations collected while flying (minimum reported great egret mean flight speed is 9.9 m s-1) [9]. The bathymetry of Tomales Bay is generally gradual except where tidal channels cut through shallow flats. Because our water depth variable represented the average depth over a 10 m^2^ area, raster cells along the edges of these channels were occasionally assigned depths deeper than great egrets can forage in [9] but still provided some accessible foraging depth. We excluded points with assigned depths greater than 1 m because we felt that these points, although biologically plausible, had too strong an influence on the models we fitted. Because there were relatively few shellfish aquaculture raster cells with depths < -0.5 m (i.e., 0.5 m above the tidal level; S1 Fig), we also restricted our analysis of habitat selection to depths ≥ -0.5 m.

Dynamic acceleration is the acceleration an object experiences due to movement, rather than gravity (static acceleration). In biologging studies that employ accelerometers, Overall Dynamic Body Acceleration (ODBA) is the sum of dynamic acceleration across the X, Y and Z axes and is a useful index of energy expenditure across a range of vertebrate taxa [33–35]. We calculated ODBA by taking the difference between static and dynamic acceleration for each of the three accelerometer axes, with the static acceleration being estimated as the average value across the entire 6 sec burst. We summed the absolute values of these differences for the entire burst for each axis and, finally, summed the acceleration for all three axes [35, 36].

### Analysis

In studies of habitat selection, how “available” habitats are defined for individual animals can have substantial influence on the inference that can be made [37, 38]. Integrated Step Selection Analysis (iSSA) allows simultaneous inference about both habitat selection processes and movement processes [39]. In iSSA, analysis is based on “steps” representing sequential locations separated by equal time intervals. At the starting point of each step, a particular area of habitat is available for the animal to select from, based on the movement capacity of that animal. The location of the animal at the end point of each step is paired with a set of “available” points that are randomly generated from the distributions of lengths and turn angles of all steps by that animal. Thus, each animal’s observed movement characteristics (step length and turn angle) determines which habitat is considered available from the starting location of each step, and each starting location has its own unique domain of available habitat. This reduces some bias in arbitrary, investigator defined availability domains, and it allows treatment of each observed step (and associated control steps) as the level of measurement while reducing the effect of lack of independence on standard errors [39, 40].

GPS sampling error can contribute substantially to estimated distance travelled by tagged animals [41], particularly as the step length travelled during the sampling interval approaches the magnitude of that measurement error [42]. Although we had 5-min-interval GPS data, preliminary data summarization indicated that a sampling interval of 10 minutes yielded step lengths longer than our GPS tag sampling error and avoided an excessive number of steps with no movement, but was a sufficiently short time span to evaluate fine scale habitat selection. We generated 10 random steps (yielding a balance between estimation error and computational burden [39]) for each observed step and extracted habitat characteristics at the start and end of each observed and available step. For all parts of our analysis, it is important to keep in mind that inference was dependent on both the sampling interval (time between locations) and the spatial resolution at which we considered habitat attributes. For example, increasing the sampling interval (and thus expected distance travelled on each step) would expand the radius of the availability domain, whereas a coarser resolution habitat raster may have limited the diversity of wetland types available at a given sampling interval. Additionally, the water depths we calculate are not necessarily the precise water depths that egrets were selecting at each GPS timestamp. Rather, these depths represented the average depth across the 10 m^2^ raster cell within which the GPS point was located. We chose to use 10 m habitat raster resolution to match the accuracy of our GPS tags. We did not explore the sensitivity of our results to variation in sampling interval or habitat raster resolution.

We were interested in examining third order resource selection (selection of feeding sites), and this selection was conditional on tagged egrets first selecting to live in the San Francisco area (first order selection) and then selecting to forage at Tomales Bay (second order selection) [43]. We fitted seven conditional logistic regression models to the data for each individual egret, to test our hypotheses about habitat selection. The response of these models was whether a location was “used” or “available.” Because iSSA is focused on movement steps, one can choose to evaluate wetland type at the start or the end of the step. We used wetland type at the end of the step for inference about habitat selection [44]. We fitted iSSA models to each individual egret separately rather than using bird id as a random effect [39, 44]. To test our prediction of lower selection for shellfish aquaculture areas than for natural wetlands, we fitted a “full” model with the interaction between the 4-level wetland type variable and a quadratic effect for time-specific water depth (i.e., to allow selection of each wetland type to vary differently as water depth changed). We did not consider additional possible predictor variables (e.g., time of year, sex of bird, etc.) because of the small number of birds in our study. To evaluate evidence for the effects of wetland type and water depth on selection, we compared AICc values (Akaiki Information Criterion corrected for small sample size) [45] between this full model and the six nested models that represent the possible combinations between these variables, including the linear and quadratic effects of water depth (candidate models shown in S1 Table). We calculated relative selection strength from the coefficients in the best-supported (lowest AICc value) of these models.

We used a combination of the conditional logistic regression models described above and linear mixed effect models to evaluate evidence for differences in foraging behavior between wetland types. To evaluate wetland-based variation in step lengths we added the interactions between wetland type at the start of the step and both step length and the natural logarithm of step length [44, 46] to the best supported habitat selection model. We judged this model’s ability to explain variability in our data by comparing its AICc value to the best-supported model from the habitat selection analysis. We then used the estimated coefficients for step lengths to modify the naïve step length estimates (from the raw data) to remove the effect of habitat selection from our estimate of movement differences between wetland types [44]. We used linear mixed models to evaluate whether ODBA varied between habitats, and we used ODBA values collected at the original 5 min interval to maximize available data. This model included a random effect for bird id, and therefore model estimates were taken as our estimate across all tagged birds. We fitted a model with wetland type as the predictor variable and compared it to an intercept-only model with the Likelihood Ratio Test to evaluate evidence for wetland type-based differences in ODBA. We fitted these models with Maximum Likelihood rather than Restricted Maximum Likelihood because we were interested in evaluating the importance of the fixed effects [47]. As with the habitat selection part of our analysis, we did not consider other possible variables that might affect foraging behavior (e.g., sex, time of year) because of the small number of birds in our sample.

Where our best supported models included interaction terms, we base interpretation of results on plotted effects rather than coefficients for individual predictor variables. We used ArcGIS Pro v2.4.0 [48] and R version 4.0.2 [49] for spatial data processing, and R for all analysis. We used the function predict.tidem from the R package oce [50] to interpolate tide level values at each GPS timestamp. We used the R package amt [44] to process GPS data and analyze habitat selection and step length. We used the package lme4 [51] for linear mixed models to analyze ODBA. Code files are archived here: https://doi.org/10.5281/zenodo.5571072. Tagging data are archived here: https://www.movebank.org/cms/webapp?gwt_fragment=page=studies,path=study247850178.

## Results

We obtained a mean = 177 ± SE 31 days of Tomales Bay foraging data per tagged great egret, collected between 10 June 2017 and 31 July 2020 (Table 1). Although we had data for 10 egrets using Tomales Bay, we excluded 3 from formal modelling. These birds almost completely avoided using shellfish aquaculture areas (Table 1), which led to convergence issues when maximizing likelihood for the conditional logistic regression models. Some tagged birds spent time outside the study area during the study period. For all tagged birds for which we ceased receiving data, we were unable to determine whether the cause was death, tag loss or tag failure.

**Table 1:** Summary of tagged great egrets.

		days tracked on Tomales Bay		number of steps by end location				
Bird ID	capture site	total days	date range	eelgrass	shellfish	tidal marsh	other tidal	total steps
GREG_10	CG	360	Sep 21, 2018; Jul 31, 2020	4074	100	1116	7420	12710
GREG_8	TP	343	Jul 23, 2018; Jun 30, 2020	3874	386	414	5417	10091
GREG_2	TP	188	Jun 10, 2017; Jun 22, 2018	1463	1038	2029	4006	8536
GREG_1	TP	182	Jun 10, 2017; Aug 31, 2018	2545	672	666	3167	7050
GREG_3	TP	113	Jun 11, 2017; Jun 22, 2018	1672	882	1754	2016	6324
GREG_6	WC	123	Jun 12, 2018; Aug 17, 2019	1740	674	641	2708	5763
GREG_11*	CG	173	Mar 26, 2019; Jul 9, 2020	2078	3	754	2417	5252
GREG_7*	CG	144	Jul 5, 2018; Dec 12, 2018	978	6	593	1822	3399
GREG_5	TP	73	Jun 8, 2018; Feb 7, 2019	1288	199	111	1588	3186
GREG_9*	CG	77	Sep 19, 2018; Dec 4, 2018	275	2	807	1944	3028

Number of days tracked on Tomales Bay, CA and number of steps ending in each wetland type for GPS tagged great egrets. Capture sites are as follows: CG = Cypress Grove; TP = Toms Point; WC = Walker Creek. Models failed to converge for birds with few observed steps in shellfish aquaculture areas (indicated by *), so these egrets were excluded from statistical analysis. Total number of days is less than the number of days comprising the date range for egrets that left the study area for migration or other movements.

For our first objective, testing the relative selection of natural wetlands vs. shellfish aquaculture areas, there was consistency among birds in the best-supported models. For all birds, the model with the interaction between wetland type and the quadratic effect of water depth was the best supported and the one with the main effects only of habitat and quadratic water depth was the second best supported. The difference in AICc values (Δ AICc) for these second-ranked models were large (69–229), indicating strong evidence that depth-based resource selection took a substantially different form among wetland types. Thus, we based inference on the best models only (AICc values for all models presented in S1 Table). Recall that the water depth variable represented the average depth over a 10 m^2^ area where each GPS point was located, rather than the precise depth the bird was standing in at the GPS timestamp.

**Fig 2:**
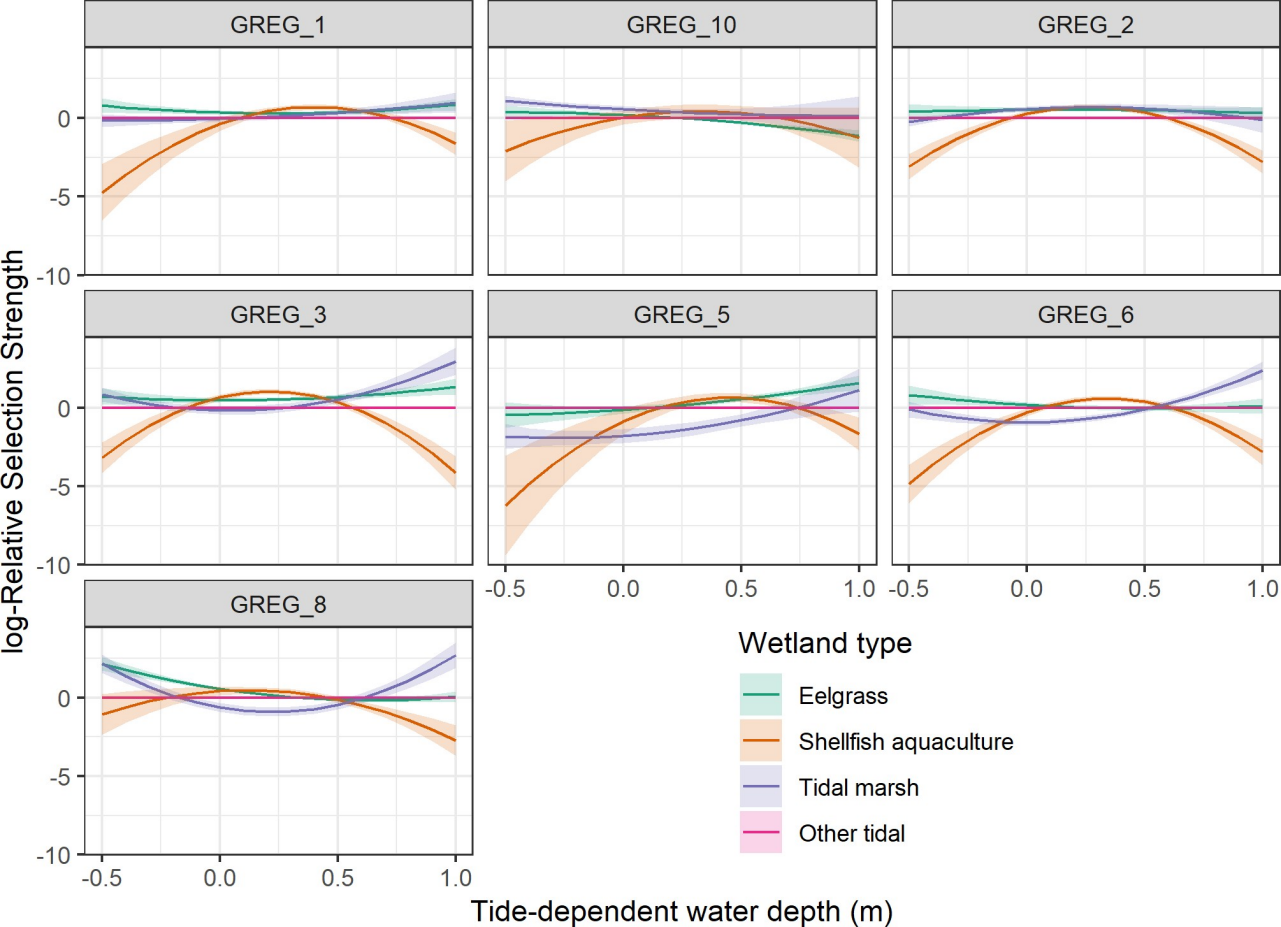
log-Relative selection strength and 95% confidence intervals of different tidal wetland types by seven GPS tagged great egrets at Tomales Bay, CA, 2017–2020, while accounting for tide-based changes in water depth. Other tidal areas (the reference level) were mostly unvegetated inter- and subtidal mud flats. Water depth represents the average depth in the 10 m^2^ grid cell around where the bird was located. Negative values of water depth indicate the bird was located above predicted water level.

Plotted estimates of log-Relative selection strength from the best-supported models show a degree of individual variation in habitat selection, but also some consistent patterns shared among all or most birds (Fig 2). The most-consistent pattern, observed across all egrets, was a strong quadratic effect (concave downward) of water depth on selection of shellfish aquaculture areas. Between depths of approximately 0 m to 0.6 m, great egrets selected shellfish aquaculture areas about as strongly as eelgrass areas. For two birds (GREG_3 and GREG_6) selection was stronger for shellfish aquaculture areas than eelgrass (with non-overlapping 95% CI) in a very narrow depth band around 0.25 m to 0.5 m. However, for most birds selection for shellfish aquaculture areas was lower than for eelgrass (with non-overlapping 95% CI) when water was deeper than about 0.5 m and in areas that were above the tide line (Fig 2). This result was not simply a factor of shellfish aquaculture infrastructure existing in a narrower, intermediate elevation than natural wetlands, although this may have contributed somewhat at the most negative depths (S1 Fig); the depth range of higher selection for shellfish aquaculture areas was much narrower than the overall depth range we observed for shellfish aquaculture areas (see Discussion for further detail). We also observed a quadratic effect of water depth on selection for eelgrass and tidal marsh, but this effect was generally much weaker than we observed for shellfish aquaculture areas and the quadratic shape varied more among egrets (Fig 2). We found some evidence for differences in selection strength among the three natural wetland types. Eelgrass was selected more strongly than other tidal (mostly unvegetated flats) for most egrets across most depths. For some egrets selection was strongest for tidal marsh when those areas were deeply flooded by higher tides (e.g. GREG_6; Fig 2).

**Fig 3:**
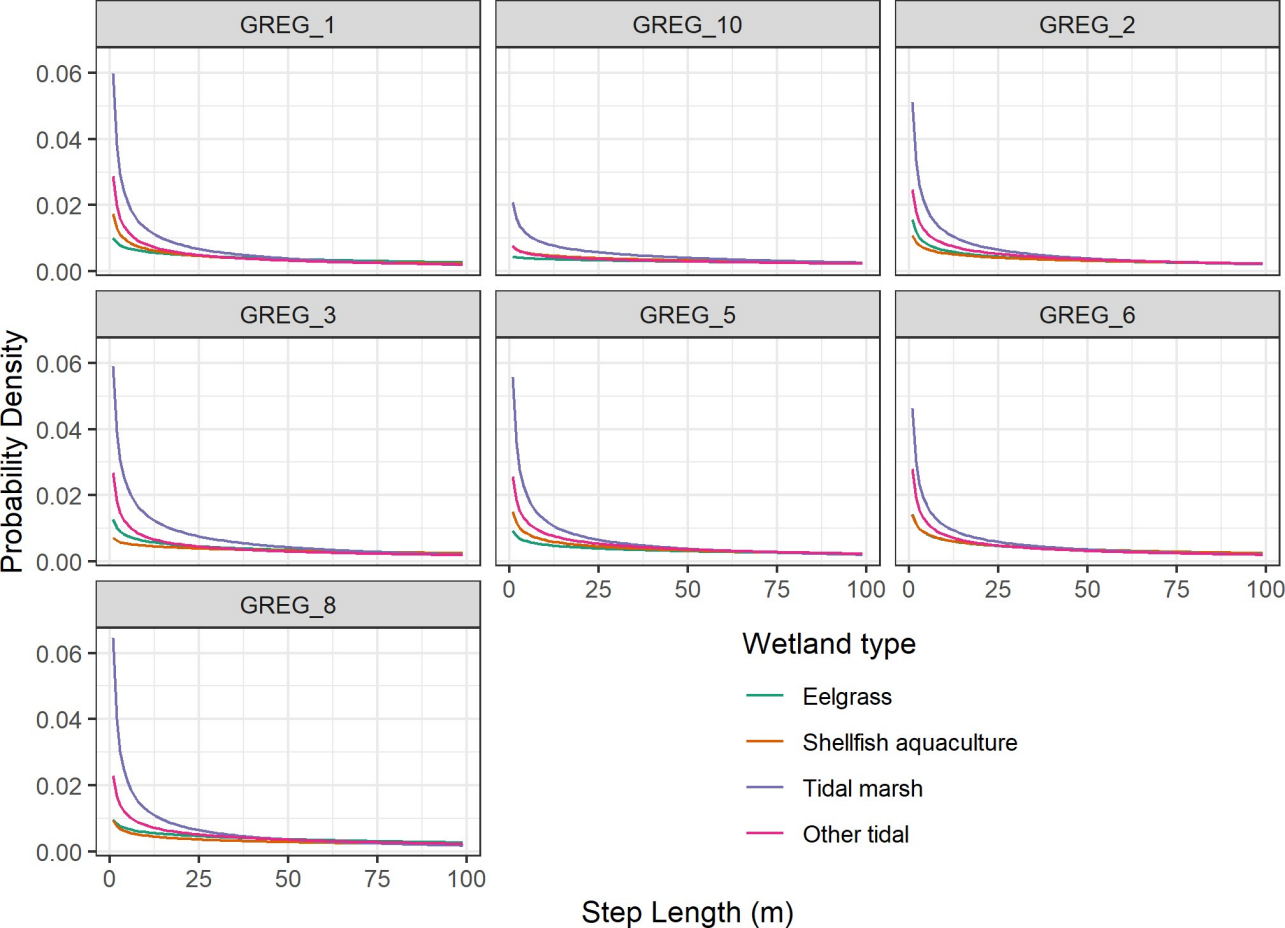
Distribution of step lengths (straight distance between consecutive evenly spaced timestamps) in different tidal wetland types by foraging great egrets at Tomales Bay, CA, 2017–2020. Probability density was calculated for step lengths 0–400 m, but only step lengths where there was visible difference in plotted values among habitats are shown.

For our second objective, describing foraging movement and behavior, we found differences among wetland types, but they mostly involved tidal marsh being different than the other wetlands. In our investigation of step length, the model with the step length*habitat interaction received more support than the one without those variables for all birds (Δ AICc for habitat selection models = 129–601), providing some evidence for consistent differences in step length among all tagged egrets (S2 Table). The plotted probability density of step length distributions, once adjusted for movement related to habitat selection, showed that step length in all wetland types were heavily skewed toward shorter step lengths (Fig 3). However, this skewing was much stronger (i.e., greater probability density for shorter steps) for tidal marsh than other wetlands for all birds.

**Fig 4:**
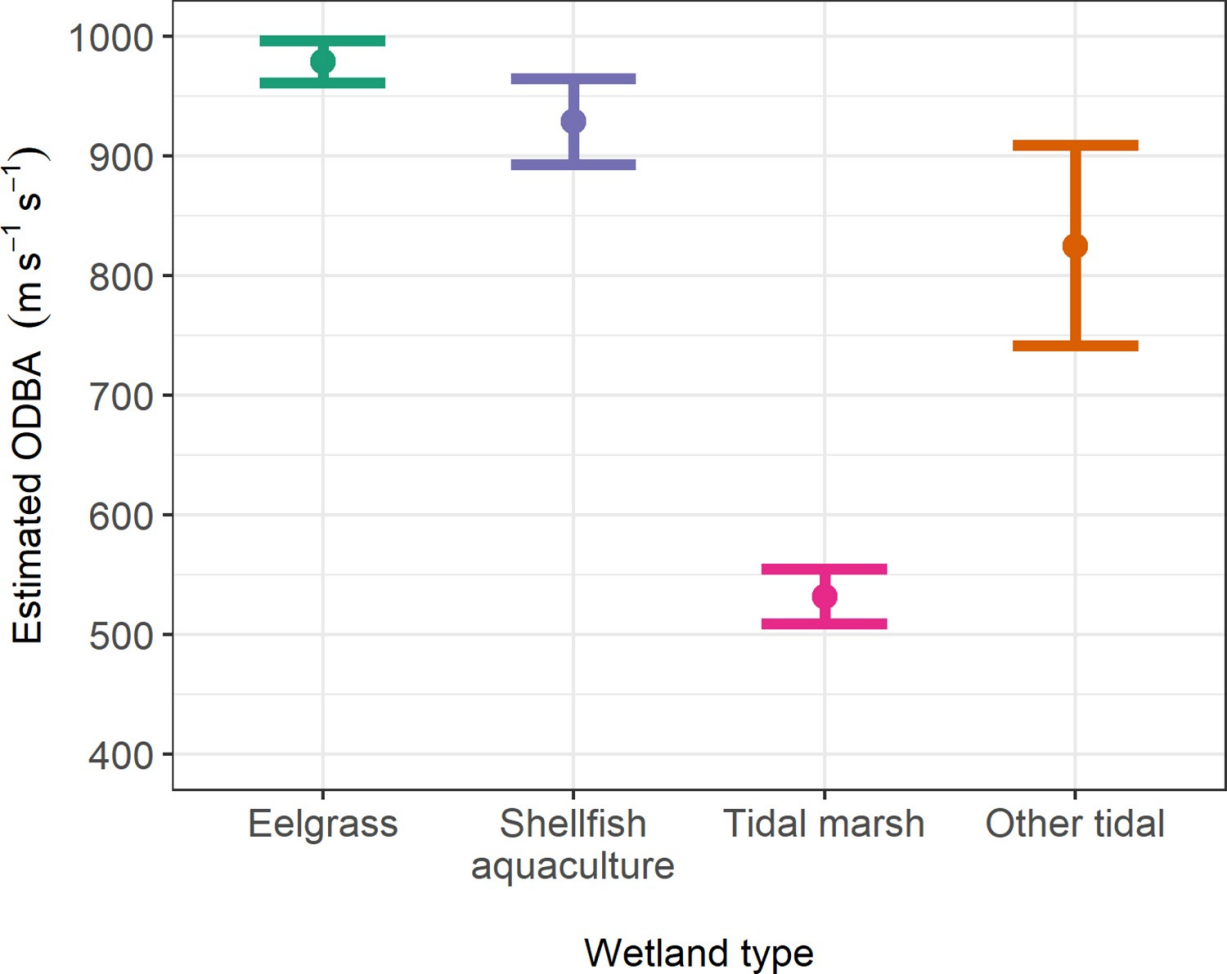
Overall dynamic body acceleration (ODBA) by seven GPS tagged great egrets in different tidal wetland types at Tomales Bay, CA, 2017–2019. Estimates and 95% profile likelihood confidence intervals are from a linear mixed effect model with bird ID included as a random effect.

In our test for different ODBA among wetland types, the model with wetland type provided a substantially better fit to the data than did the intercept only model (χ^2^ (3) = 1249, p < 0.001). The estimated coefficient for tidal marsh was strongly negative and with 95% CI that substantially excluded zero (β = -292.4, 95% CI -315.6 to -269.2). The coefficients for eelgrass and shellfish aquaculture areas were both positive and with 95% CI not overlapping zero (eelgrass β = 154.5, 95% CI 136.7 to 172.2; shellfish β = 104.3, 95% CI 68.5 to 140.2). Thus, there was good evidence that ODBA was different among eelgrass, shellfish aquaculture areas and other tidal, and convincing evidence that ODBA in tidal marsh was different than the other 3 wetland types. ODBA was greatest for eelgrass and shellfish aquaculture areas (about 50 ms^-1^s^-1^ greater for the former), intermediate for other tidal wetlands (the reference level) and was substantially lower in tidal marsh than the other three wetland types (Fig 4).

## Discussion

In this first investigation of great egret foraging behavior and habitat selection in areas where shellfish aquaculture operations exist, we found support for our prediction of greater selection for eelgrass than for shellfish aquaculture areas. Three of 10 egrets effectively avoided shellfish aquaculture areas. Among the egrets that did forage in shellfish aquaculture areas, habitat selection was contingent on tide-based water depth. Specifically, great egrets were more likely to select eelgrass than shellfish aquaculture areas across most water depths, suggesting lower use or avoidance of aquaculture areas at some times through the range of the tidal cycle. We also found that step lengths while foraging were similar among all wetlands except tidal marsh, where they were shorter. Energy expenditure was greatest in eelgrass and shellfish aquaculture areas, intermediate other (mostly unvegetated) tidal areas, and substantially lower in tidal marsh than the former three wetland types. We believe these results for tidal marsh partially reflect short-term roosting there between foraging bouts.

Because we acquired data for only 10 birds, and used data from only seven for formal modelling, our results effectively describe the foraging responses to shellfish aquaculture by the subject individuals but may not accurately predict the behaviors of other individuals in other locations or times. We did not find consistent support among all egrets for our prediction that eelgrass would be selected more strongly than other natural wetlands. Because of our small sample, we have most confidence in the observed pattern that was consistent among all egrets (the difference between shellfish aquaculture and natural wetlands as a group), and we don’t discuss further the inconsistent patterns of selection among natural wetlands.

Three out of ten egrets avoided shellfish aquaculture areas almost entirely and were excluded from formal modelling, a pattern that may suggest these three birds did not perceive the shellfish aquaculture areas as valuable foraging habitat. Similarly, shorebirds (Suborders *Charadrii* and *Solopaci*) at Tomales Bay generally avoid shellfish aquaculture areas, although Willet (*Tringa semipalmata*) appears an exception [52]. The three egrets that avoided shellfish aquaculture areas were captured at Cypress Grove, approximately 5 km from the Walker Creek delta where most of the shellfish aquaculture placed in egret-accessible depths is located on Tomales Bay (Fig 1). Most other birds were captured at Toms Point or Walker Creek, within 0.3–1.5 km of these main shellfish aquaculture areas. We cannot rule out that this apparent avoidance of shellfish aquaculture areas was due to some unrelated bay-wide segregation of foraging areas, and not directly due to avoidance of shellfish aquaculture. However, great egrets in the San Francisco Bay area regularly fly up to 10 km from colonies to forage [53], so reaching the shellfish aquaculture areas was well within the flight capabilities and foraging flight distances of egrets captured at all 3 trapping locations. Indeed, two of these Cypress Grove-captured birds (GREG_7 and GREG_11) repeatedly used areas around Walker Creek delta, but nevertheless still mostly avoided shellfish aquaculture areas. The fourth Cypress Grove-captured bird also regularly foraged around Walker Creek and did not avoid shellfish aquaculture areas.

We found that time- and location-specific changes in water depth (due to tidal action) were an important component in how great egret habitat selection varied between shellfish aquaculture areas and natural wetlands. Relative selection for shellfish aquaculture areas showed a strong quadratic response across the range of water depths we investigated, whereas selection for natural wetlands was generally constant across that range. Selection of shellfish aquaculture areas reached the strength of selection for natural wetlands only in a narrow depth range, but relative selection of shellfish aquaculture areas did not exceed that for natural wetlands at any water depth. Great egrets appear quite capable of ascertaining the relative benefits and costs of foraging in different areas that result from variance in prey density, prey capturability, and competition [16, 54, 55]. Generally, the density of foraging great egrets is greatest when water depths are between 20–40 cm [9]. Based on our results, shellfish aquaculture areas appear to provide foraging opportunities only in this narrow, preferred range of water depths, whereas natural habitats provide more diverse foraging opportunities across a broader range of water depths.

Although the four wetland types we considered span slightly different depth ranges at Tomales Bay, our results for habitat selection are not simply an artifact of aquaculture infrastructure occurring in a narrower, intermediate elevation range than the other wetland types (and thus experiencing a narrower range of water depths). We restricted our analysis to depths with sufficient representation among all wetland types, and the quadratic pattern of selection we observed was apparent across the range of depths that shellfish aquaculture areas experienced. Given the opportunity to forage in adjacent shellfish aquaculture areas or natural wetlands, the egrets in our study selected natural wetlands more strongly when water depths were greater than about 0.5 m and in areas that were exposed above the tide line. Because depths > 0.5 m are approaching the deepest water egrets regularly forage in [9], it is also important to recall our scale of inference when interpreting our results. Our water depth variable represented the average depth in the 10 m^2^ area around where each GPS location was recorded, not the specific depth of water the egret was standing in. Where our models indicated greater selection for natural wetlands than shellfish aquaculture areas in water deeper than 0.5 m, we interpret these results to indicate that the egrets were more likely to probe the limits of their foraging depth (i.e., search for and use small areas of shallower water in deeper areas) in natural wetlands than in shellfish aquaculture areas. It may also be that the natural wetlands contained more small-scale heterogeneity in depths than did shellfish aquaculture areas.

Water depth, and especially temporal change in depth, is an important component of great egret foraging in other places it has been investigated. For instance, in the Everglades, great egrets select for areas where patterns of flooding and water drawdown operate at multiple spatiotemporal scales (e.g. daily and weekly tide patterns and seasonal climatic patterns) and act to concentrate prey [18, 56–58]. In tidal systems in southern Florida, time-integrated habitat availability (due to tidal cycles) was the resource attribute with the strongest effect on probability of use by wading birds across all habitats investigated [54]. The intertidal and shallow subtidal areas of Tomales Bay are characterized by subtle heterogeneity in the substrate surface, and egrets foraging above the tide line often seem to be focusing on small tidal puddles (pers. obs.). It is likely that shallow depressions in the intertidal areas serve as hydrologic refugia for egret prey during lower tides, and that egrets respond to the higher prey densities there. The quadratic pattern of selection for shellfish aquaculture areas may be driven by the interaction of prey density and prey availability. Although prey density may be greater in areas with thicker vegetation, a combination of intermediate vegetation thickness and shallower water depth may yield better prey capture by wading birds [16]. On Tomales Bay, these conditions may have been mimicked by shellfish aquaculture equipment as the tides rose and fell through it. The resulting shallow depths and moderate cover may have concentrated aquatic prey or made their escape responses slower or otherwise reduced, or some combination of both.

Tagged great egrets expended less energy and had shorter step lengths in tidal marsh than the other wetland types we considered. Egrets often roost in Tomales Bay tidal marsh areas between periods of active foraging (pers. obs.), as well as forage there, so these results may partially reflect this behavior. We found only slight differences in foraging behavior between natural wetlands and shellfish aquaculture areas. The cost of flying for great egrets has been estimated to be much greater than that of foraging [12]. If egrets are flushed more frequently in shellfish aquaculture areas during harvest or other maintenance activities, compared to flush rates in eelgrass, then the difference between energy acquisition and energy expenditure may differ between these wetland types despite the similar values of ODBA. Additionally, differences in prey type between shellfish aquaculture areas and natural wetlands (which we did not examine) may also lead to differences in energy acquisition despite similar energy expenditure. Based on our results we believe that a valuable next investigation would be to test the hypothesis that prey capture and energy acquisition are equal among shellfish aquaculture areas and natural wetlands.

### Management implications

It appears that the current arrangement of eelgrass beds, tidal marsh, shellfish aquaculture areas, and mud flat provide a diversity of foraging opportunities to egrets across the tidal range. Although we did not directly quantify energy gain in each wetland type, our results suggest that shellfish aquaculture areas were perceived or experienced by these tagged great egrets as providing lower foraging quality than eelgrass or tidal marshes; however foraging studies explicitly addressing energetics are needed to evaluate this. Since eelgrass is federally designated in the U.S. as Essential Fish Habitat and a Habitat of Particular Concern, and managing agencies have adopted a “no net loss” policy [23], it is unlikely that any potential expansion of shellfish aquaculture in Tomales Bay will directly reduce availability of eelgrass to foraging great egrets. However, further conversion of unvegetated tidal areas in Tomales Bay for shellfish aquaculture may nevertheless reduce the amount of time that those areas provide suitable foraging opportunities for great egrets across the entire range of tidal depths. If this causes egrets to spend more time foraging in eelgrass, then this may change the nature of top down predation pressure in eelgrass systems [24].

Sea level rise associated with climate change may alter the overall foraging opportunities for great egrets on Tomales Bay, as both eelgrass and shellfish aquaculture are forced to migrate upslope. If eelgrass cannot migrate upslope to match the pace of sea level rise, the overall availability, and use, of eelgrass by egrets may decline. Thus, management actions that encourage upslope expansion of eelgrass beds to match sea level rise seem likely to benefit great egrets. In addition, such conservation efforts, which are likely to be critical in sustaining the substantial, broader conservation values of dwindling eelgrass beds [23], could conflict dramatically with any landward movement of intertidal shellfish growing areas needed to sustain the viability of shellfish aquaculture.

## Supporting information

S1 Fig Elevations for each wetland type. Density of used and available points for each wetland type across the range of depths considered, for investigating habitat selection by GPS-tagged great egrets at Tomales Bay, CA, 2017–2020. Lines represent density of available and used points at each depth for each wetland type. Negative depth values indicate locations above the predicted water level.
(TIF)

S1 Table Habitat selection model selection. Model selection results for evaluating differences in foraging habitat selection among wetland habitat types, while accounting for water depth, by GPS-tagged great egrets at Tomales Bay, CA, 2017–2020. K is the number of parameters, Δ AICc is the difference in AICc value between the top model and the current model, and AICc Wt. is the AICc model weight.
(DOCX)

S2 Table Step length model selection. Model selection results for evaluating differences in foraging step length among wetland habitat types, while accounting for water depth, by GPS-tagged great egrets at Tomales Bay, CA, 2017–2020. K is the number of parameters, Δ AICc is the difference in AICc value between the top model and the current model, and AICc Wt. is the AICc model weight.
(DOCX)
