# Supplementary figures and images for: Great egret (Ardea alba) habitat selection and foraging behavior in a temperate estuary: Comparing natural wetlands to areas with shellfish aquaculture
Source: PLoS One. 2021 Dec 31;16(12):e0261963. doi: 10.1371/journal.pone.0261963 (PMC8719746; doi:10.1371/journal.pone.0261963)

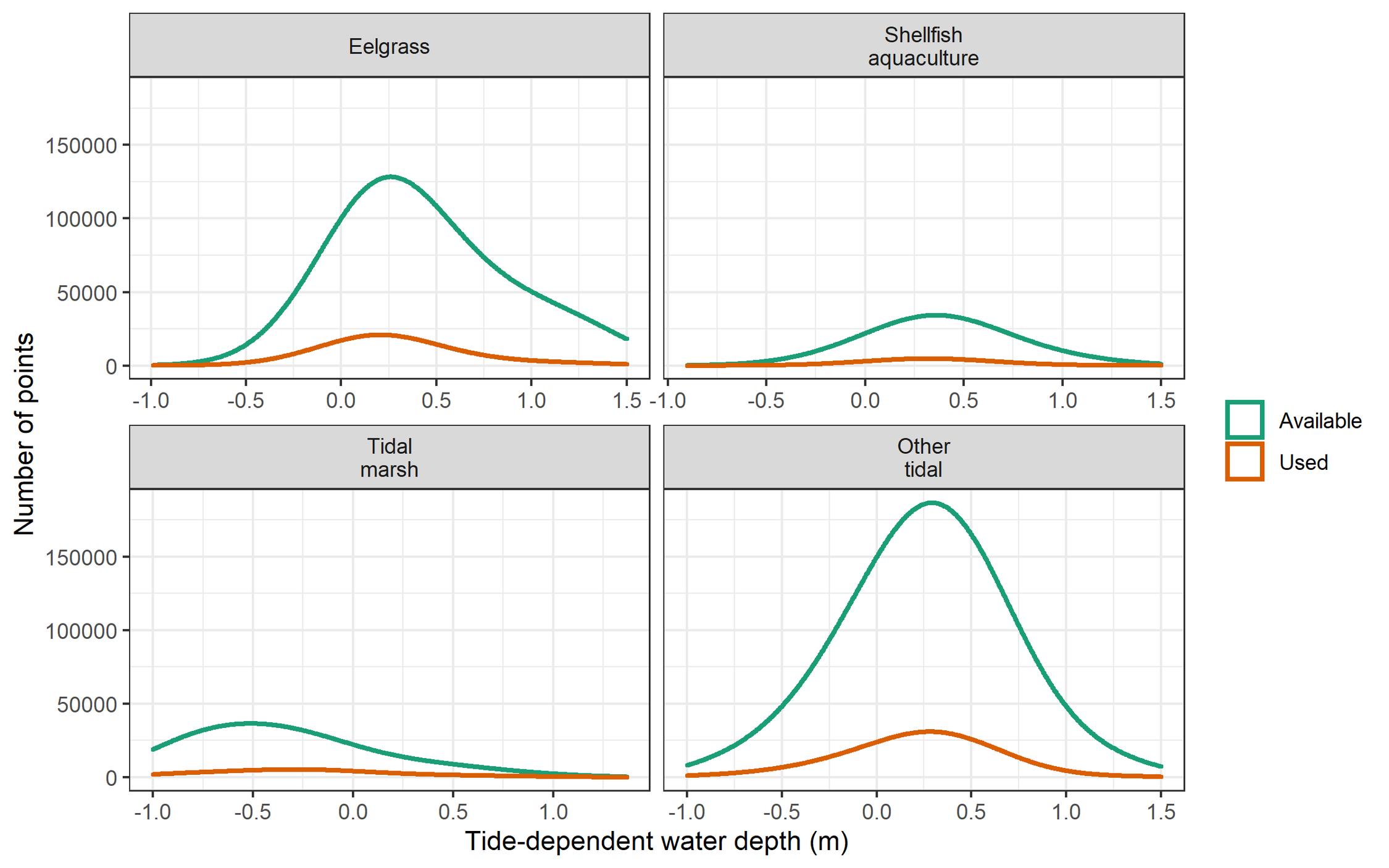

Supplement: S1 Fig — Density of used and available points for each wetland type across the range of depths considered, for investigating habitat selection by GPS-tagged great egrets at Tomales Bay, CA, 2017–2020. Lines represent density of available and used points at each depth for each wetland type. Negative depth values indicate locations above the predicted water level. (TIF) [file pone.0261963.s001.tif]
