# Supplementary material for: Great egret (Ardea alba) habitat selection and foraging behavior in a temperate estuary: Comparing natural wetlands to areas with shellfish aquaculture
Source: PLoS One. 2021 Dec 31;16(12):e0261963. doi: 10.1371/journal.pone.0261963 (PMC8719746; doi:10.1371/journal.pone.0261963)
Supplement: S1 Table — Model selection results for evaluating differences in foraging habitat selection among wetland habitat types, while accounting for water depth, by GPS-tagged great egrets at Tomales Bay, CA, 2017–2020. K is the number of parameters, Δ AICc is the difference in AICc value between the top model and the current model, and AICc Wt. is the AICc model weight. (DOCX) [file pone.0261963.s002.docx]

S1 Table

| Bird ID | model structure | K | Δ AICc | AICc Wt. | ln(likelihood) |
| --- | --- | --- | --- | --- | --- |
| GREG_1 | wetland type * depth^2^ | 14 | 0.0 | 1 | -12,066.952 |
|  | wetland type + depth^2^ | 8 | 69.6 | 0 | -12,107.778 |
|  | depth^2^ | 5 | 133.2 | 0 | -12,142.564 |
|  | wetland type * depth | 10 | 450.9 | 0 | -12,296.414 |
|  | wetland type + depth | 7 | 583.0 | 0 | -12,365.444 |
|  | depth | 4 | 643.2 | 0 | -12,398.571 |
|  | wetland type | 6 | 817.3 | 0 | -12,483.629 |
| GREG_2 | wetland type * depth^2^ | 14 | 0.0 | 1 | -13,748.529 |
|  | wetland type + depth^2^ | 8 | 116.6 | 0 | -13,812.851 |
|  | depth^2^ | 5 | 238.4 | 0 | -13,876.750 |
|  | wetland type * depth | 10 | 411.9 | 0 | -13,958.467 |
|  | wetland type + depth | 7 | 542.3 | 0 | -14,026.665 |
|  | depth | 4 | 614.8 | 0 | -14,065.929 |
|  | wetland type | 6 | 752.0 | 0 | -14,132.507 |
| GREG_3 | wetland type * depth^2^ | 14 | 0.0 | 1 | -8,638.085 |
|  | wetland type + depth^2^ | 8 | 160.5 | 0 | -8,724.362 |
|  | depth^2^ | 5 | 284.4 | 0 | -8,789.265 |
|  | wetland type * depth | 10 | 431.2 | 0 | -8,857.704 |
|  | wetland type + depth | 7 | 501.9 | 0 | -8,896.023 |
|  | depth | 4 | 613.5 | 0 | -8,954.846 |
|  | wetland type | 6 | 702.9 | 0 | -8,997.558 |
| GREG_5 | wetland type * depth^2^ | 14 | 0.0 | 1 | -4,^2^.525 |
|  | wetland type + depth^2^ | 8 | 83.3 | 0 | -4,270.189 |
|  | wetland type * depth | 10 | 135.2 | 0 | -4,294.108 |
|  | wetland type + depth | 7 | 177.1 | 0 | -4,318.104 |
|  | depth^2^ | 5 | 179.5 | 0 | -4,321.288 |
|  | depth | 4 | 312.1 | 0 | -4,388.577 |
|  | wetland type | 6 | 433.2 | 0 | -4,447.136 |
| GREG_6 | wetland type * depth^2^ | 14 | 0.0 | 1 | -10,351.019 |
|  | wetland type + depth^2^ | 8 | 221.8 | 0 | -10,467.941 |
|  | depth^2^ | 5 | 231.5 | 0 | -10,475.780 |
|  | wetland type * depth | 10 | 556.4 | 0 | -10,633.200 |
|  | wetland type + depth | 7 | 762.6 | 0 | -10,739.317 |
|  | depth | 4 | 795.1 | 0 | -10,758.580 |
|  | wetland type | 6 | 923.5 | 0 | -10,820.748 |
| GREG_8 | wetland type * depth^2^ | 14 | 0.0 | 1 | -15,645.492 |
|  | wetland type + depth^2^ | 8 | 229.0 | 0 | -15,766.017 |
|  | depth^2^ | 5 | 254.1 | 0 | -15,781.528 |
|  | wetland type * depth | 10 | 1,116.5 | 0 | -16,207.734 |
|  | wetland type + depth | 7 | 1,492.8 | 0 | -16,398.911 |
|  | depth | 4 | 1,551.1 | 0 | -16,431.045 |
|  | wetland type | 6 | 1,690.3 | 0 | -16,498.632 |
| GREG_10 | wetland type * depth^2^ | 14 | 0.0 | 1 | -14,422.526 |
|  | wetland type + depth^2^ | 8 | 86.3 | 0 | -14,471.673 |
|  | depth^2^ | 5 | 136.2 | 0 | -14,499.642 |
|  | wetland type * depth | 10 | 1,191.1 | 0 | -15,022.096 |
|  | wetland type + depth | 7 | 1,793.5 | 0 | -15,326.287 |
|  | depth | 4 | 1,818.0 | 0 | -15,341.530 |
|  | wetland type | 6 | 2,022.0 | 0 | -15,441.509 |
