# Supplementary material for: Great egret (Ardea alba) habitat selection and foraging behavior in a temperate estuary: Comparing natural wetlands to areas with shellfish aquaculture
Source: PLoS One. 2021 Dec 31;16(12):e0261963. doi: 10.1371/journal.pone.0261963 (PMC8719746; doi:10.1371/journal.pone.0261963)
Supplement: S2 Table — Model selection results for evaluating differences in foraging step length among wetland habitat types, while accounting for water depth, by GPS-tagged great egrets at Tomales Bay, CA, 2017–2020. K is the number of parameters, Δ AICc is the difference in AICc value between the top model and the current model, and AICc Wt. is the AICc model weight. (DOCX) [file pone.0261963.s003.docx]

S2 Table

| Bird ID | model structure | K | Δ AICc | AICc Wt. | ln(likelihood) |
| --- | --- | --- | --- | --- | --- |
| GREG_1 | wetland type * step length | 20 | 0.0 | 1 | -12,499.216 |
|  | wetland type * depth^2^ | 14 | 423.3 | 0 | -12,716.888 |
| GREG_2 | wetland type * step length | 20 | 0.0 | 1 | -16,597.990 |
|  | wetland type * depth^2^ | 14 | 363.7 | 0 | -16,785.850 |
| GREG_3 | wetland type * step length | 20 | 0.0 | 1 | -11,726.058 |
|  | wetland type * depth^2^ | 14 | 601.1 | 0 | -12,032.630 |
| GREG_5 | wetland type * step length | 20 | 0.0 | 1 | -4,439.276 |
|  | wetland type * depth^2^ | 14 | 129.4 | 0 | -4,509.970 |
| GREG_6 | wetland type * step length | 20 | 0.0 | 1 | -11,050.044 |
|  | wetland type * depth^2^ | 14 | 169.3 | 0 | -11,140.700 |
| GREG_8 | wetland type * step length | 20 | 0.0 | 1 | -16,586.095 |
|  | wetland type * depth^2^ | 14 | 361.9 | 0 | -16,773.035 |
| GREG_10 | wetland type * step length | 20 | 0.0 | 1 | -16,766.456 |
|  | wetland type * depth^2^ | 14 | 140.6 | 0 | -16,842.749 |
