# Supplementary material for: Great egret (Ardea alba) habitat selection and foraging behavior in a temperate estuary: Comparing natural wetlands to areas with shellfish aquaculture
Source: PLoS One. 2021 Dec 31;16(12):e0261963. doi: 10.1371/journal.pone.0261963 (PMC8719746; doi:10.1371/journal.pone.0261963)
Supplement: Peer review history

# Peer review: Decision Letter 0

**DOI:** 10.1371/journal.pone.0261963.r001

**Authors:** Vanesa Magar

21 Jul 2021

PONE-D-21-19522

Great egret (Ardea alba) habitat selection and foraging behavior in a temperate estuary: comparing natural wetlands to areas with shellfish aquaculture

PLOS ONE

Dear Dr. Jennings,

Thank you for submitting your manuscript to PLOS ONE. After careful consideration, we feel that it has merit but does not fully meet PLOS ONE’s publication criteria as it currently stands. Therefore, we invite you to submit a revised version of the manuscript that addresses the points raised during the review process.

Please submit your revised manuscript by Sep 04 2021 11:59PM. If you will need more time than this to complete your revisions, please reply to this message or contact the journal office at plosone@plos.org. When you're ready to submit your revision, log on to https://www.editorialmanager.com/pone/ and select the 'Submissions Needing Revision' folder to locate your manuscript file.

Please include the following items when submitting your revised manuscript:

- A rebuttal letter that responds to each point raised by the academic editor and reviewer(s). You should upload this letter as a separate file labeled 'Response to Reviewers'.
- A marked-up copy of your manuscript that highlights changes made to the original version. You should upload this as a separate file labeled 'Revised Manuscript with Track Changes'.
- An unmarked version of your revised paper without tracked changes. You should upload this as a separate file labeled 'Manuscript'.

If you would like to make changes to your financial disclosure, please include your updated statement in your cover letter. Guidelines for resubmitting your figure files are available below the reviewer comments at the end of this letter.

If applicable, we recommend that you deposit your laboratory protocols in protocols.io to enhance the reproducibility of your results. Protocols.io assigns your protocol its own identifier (DOI) so that it can be cited independently in the future. For instructions see: http://journals.plos.org/plosone/s/submission-guidelines#loc-laboratory-protocols. Additionally, PLOS ONE offers an option for publishing peer-reviewed Lab Protocol articles, which describe protocols hosted on protocols.io. Read more information on sharing protocols at https://plos.org/protocols?utm_medium=editorial-email&utm_source=authorletters&utm_campaign=protocols.

We look forward to receiving your revised manuscript.

Kind regards,

Vanesa Magar, Ph.D.

Academic Editor

PLOS ONE

Journal Requirements:

When submitting your revision, we need you to address these additional requirements.

1. Please ensure that your manuscript meets PLOS ONE's style requirements, including those for file naming. The PLOS ONE style templates can be found at 

https://journals.plos.org/plosone/s/file?id=wjVg/PLOSOne_formatting_sample_main_body.pdf and 

https://journals.plos.org/plosone/s/file?id=ba62/PLOSOne_formatting_sample_title_authors_affiliations.pdf

2. Please review your reference list to ensure that it is complete and correct. If you have cited papers that have been retracted, please include the rationale for doing so in the manuscript text, or remove these references and replace them with relevant current references. Any changes to the reference list should be mentioned in the rebuttal letter that accompanies your revised manuscript. If you need to cite a retracted article, indicate the article’s retracted status in the References list and also include a citation and full reference for the retraction notice.

3. In your Methods section, please provide additional location information of the study site, including geographic coordinates for the data set if available.

4. We note that the grant information you provided in the ‘Funding Information’ and ‘Financial Disclosure’ sections do not match. 

When you resubmit, please ensure that you provide the correct grant numbers for the awards you received for your study in the ‘Funding Information’ section.

5. Thank you for stating the following in the Acknowledgments Section of your manuscript: 

"Funding came from generous individual donors to Audubon Canyon Ranch and through the continued support of its Board of Directors. None of our funders had any influence on the content of the manuscript."

We note that you have provided funding information that is not currently declared in your Funding Statement. However, funding information should not appear in the Acknowledgments section or other areas of your manuscript. We will only publish funding information present in the Funding Statement section of the online submission form. 

Please remove any funding-related text from the manuscript and let us know how you would like to update your Funding Statement. Currently, your Funding Statement reads as follows: 

"Funding came from generous individual donors to Audubon Canyon Ranch and through the continued support of its Board of Directors. None of our funders had any influence on the content of the manuscript."

Please include your amended statements within your cover letter; we will change the online submission form on your behalf.

6. Your ethics statement should only appear in the Methods section of your manuscript. If your ethics statement is written in any section besides the Methods, please move it to the Methods section and delete it from any other section. Please ensure that your ethics statement is included in your manuscript, as the ethics statement entered into the online submission form will not be published alongside your manuscript. 

7. We note that Figure(s) 1 in your submission contain [map/satellite] images which may be copyrighted. All PLOS content is published under the Creative Commons Attribution License (CC BY 4.0), which means that the manuscript, images, and Supporting Information files will be freely available online, and any third party is permitted to access, download, copy, distribute, and use these materials in any way, even commercially, with proper attribution. For these reasons, we cannot publish previously copyrighted maps or satellite images created using proprietary data, such as Google software (Google Maps, Street View, and Earth). For more information, see our copyright guidelines: http://journals.plos.org/plosone/s/licenses-and-copyright.

We require you to either (1) present written permission from the copyright holder to publish these figures specifically under the CC BY 4.0 license, or (2) remove the figures from your submission:

a. You may seek permission from the original copyright holder of Figure(s) 1 to publish the content specifically under the CC BY 4.0 license.  

We recommend that you contact the original copyright holder with the Content Permission Form (http://journals.plos.org/plosone/s/file?id=7c09/content-permission-form.pdf) and the following text:

“I request permission for the open-access journal PLOS ONE to publish XXX under the Creative Commons Attribution License (CCAL) CC BY 4.0 (http://creativecommons.org/licenses/by/4.0/). Please be aware that this license allows unrestricted use and distribution, even commercially, by third parties. Please reply and provide explicit written permission to publish XXX under a CC BY license and complete the attached form.”

Please upload the completed Content Permission Form or other proof of granted permissions as an "Other" file with your submission.

In the figure caption of the copyrighted figure, please include the following text: “Reprinted from [ref] under a CC BY license, with permission from [name of publisher], original copyright [original copyright year].”

b. If you are unable to obtain permission from the original copyright holder to publish these figures under the CC BY 4.0 license or if the copyright holder’s requirements are incompatible with the CC BY 4.0 license, please either i) remove the figure or ii) supply a replacement figure that complies with the CC BY 4.0 license. Please check copyright information on all replacement figures and update the figure caption with source information. If applicable, please specify in the figure caption text when a figure is similar but not identical to the original image and is therefore for illustrative purposes only.

The following resources for replacing copyrighted map figures may be helpful:

USGS National Map Viewer (public domain): http://viewer.nationalmap.gov/viewer/

The Gateway to Astronaut Photography of Earth (public domain): http://eol.jsc.nasa.gov/sseop/clickmap/

Maps at the CIA (public domain): https://www.cia.gov/library/publications/the-world-factbook/index.html and https://www.cia.gov/library/publications/cia-maps-publications/index.html

NASA Earth Observatory (public domain): http://earthobservatory.nasa.gov/

Landsat: http://landsat.visibleearth.nasa.gov/

USGS EROS (Earth Resources Observatory and Science (EROS) Center) (public domain): http://eros.usgs.gov/#

Natural Earth (public domain): http://www.naturalearthdata.com/

[Note: HTML markup is below. Please do not edit.]

Reviewers' comments:

Reviewer's Responses to Questions

**Comments to the Author**

1. Is the manuscript technically sound, and do the data support the conclusions?

The manuscript must describe a technically sound piece of scientific research with data that supports the conclusions. Experiments must have been conducted rigorously, with appropriate controls, replication, and sample sizes. The conclusions must be drawn appropriately based on the data presented. 

Reviewer #1: Yes

**********

2. Has the statistical analysis been performed appropriately and rigorously? 

Reviewer #1: Yes

**********

3. Have the authors made all data underlying the findings in their manuscript fully available?

The PLOS Data policy requires authors to make all data underlying the findings described in their manuscript fully available without restriction, with rare exception (please refer to the Data Availability Statement in the manuscript PDF file). The data should be provided as part of the manuscript or its supporting information, or deposited to a public repository. For example, in addition to summary statistics, the data points behind means, medians and variance measures should be available. If there are restrictions on publicly sharing data—e.g. participant privacy or use of data from a third party—those must be specified.

Reviewer #1: Yes

**********

4. Is the manuscript presented in an intelligible fashion and written in standard English?

PLOS ONE does not copyedit accepted manuscripts, so the language in submitted articles must be clear, correct, and unambiguous. Any typographical or grammatical errors should be corrected at revision, so please note any specific errors here.

Reviewer #1: Yes

**********

5. Review Comments to the Author

Please use the space provided to explain your answers to the questions above. You may also include additional comments for the author, including concerns about dual publication, research ethics, or publication ethics. (Please upload your review as an attachment if it exceeds 20,000 characters)

Reviewer #1: Line 22 insert comma after marsh

Line 79 need space between [16] and comma

Line 116 labeled with

Line 117 eelgrass shown in dark gray and , shellfish….

Line 137 Does Publicly have to be capitalized. Hyphen is not necessary

Line 138 end sentence after ...our analysis.

Line 139 Capital W to start line

Line 151 remove a after in GIS….

Line 182 I did not find that DEM was defined prior to this sentence, will your reader know what this is?

Line 185 end sentence after collected

Line 186 start line with capital T

Line 198 add space between ) and [5]

Line 206 delete dur to

Line 228 “third order selection” needs to be better defined if the concept of Johnson’s hierarchical habitat selection is used. This term has not been described either in the introduction or methods prior to this point.

L.232-233 “evaluate fine scale habitat selection but avoid an excessive number of steps with no movement” – this data thinning may result in bias: “stay-put” during foraging may suggest selection, and more residence time. You may need movement steps for non-zero step selection functions; however, the more the locations you filtered out, the more the information of preference (staying there longer) you may lose. Caution needs to be exercised here.

Line 234 add space between ) and [33]

Line 245 What were an example of the observations of foraging behavior that prompted you to select this scale, an explanation would help the reader.

Line 248 what is a small number, state how many models, be specific

L. 252-253 The time-interval that you “chose” may affect the model outcomes because birds may choose to stay longer with shorter step length if wetland types are desirable; however, a part of this information has been thrown out by filtering the data. A sensitivity analysis as the authors mentioned (L. 246-247) is critical.

Line 252-254 you chose to evaluate wetland type at the start or end of the step. Use this same order in the next sentence

Line 256 than for natural wetlands

Line 257 “interactions between the 4-level wetland type variable and time-specific water depth”: have you checked whether the wetland types and water depth are correlated before they are included in the same model?

Line 261 … we deemed a quadratic response to be the most biologically likely… based on what? How did you determine this approach was the most biologically likely?

Line 261-262 The authors decided to use quadratic functions as described in the methods section without further justification using the literature or some preliminary analysis. The relationship with other three wetland types appeared to be linear based on Fig. 2.

Line 264 add space between ) and [37]

Line 301 Table 1 do you have an explanation as to the egrets that quit (?) sending data in 2018 and 2019? Did they leave the area due to migration? If so, did they revisit the area? Did you have any transmitter failure? Did you have mortality?

Lines 320-332 To better support and clarify the statements, a box plot of tidal water depth for four wetland types (maybe as a supplementary figure) would be useful.

Line 331-332 … natural wetlands than for shellfish aquaculture areas across most water depths… define most, number, percentage? Hard to determine how important this is.

Line 372 delete they

Line 393 aquaculture areas were well within …

Lines 398-399 “…showed a strong quadratic response across the range of water depths we investigated,…” Does this suggest your model only fit data on shellfish aquaculture well, failing on the other three wetland types?

Line 459 insert comma after marsh

Overview:

This is an important study because there has been little attention to the relationships between shellfish aquaculture and wading-bird habitat selection during the past. An interesting part of this study is the quantification of foraging activities with movement speed and activity level (with accelerometer data). Their results of the analyses look fine from their reports of AICc values and Figures. However, the quadratic curve is only obvious with shellfish aquaculture in Figure 2. As the tidal water depth increased between 0 and 1, relative selection strength converged among the four types of wetlands (Fig. 2). The authors decided to use quadratic functions as described in the method section without further justification using the literature or some preliminary analysis (L.261-262). The relationship with other three wetland types appeared to be linear based on Fig. 2. To better support and clarify the statements between lines 320-332, a box plot of tidal water depth for the four wetland types (maybe as a supplementary figure) would be useful.

The actual description of their statistical analysis is good, but my main concerns are twofold. 1) sample size of seven birds is too small for a reliable inference even when analysis is by individual bird; and 2) data filtering may have affected the results and conclusions.

**********

6. PLOS authors have the option to publish the peer review history of their article (what does this mean?). If published, this will include your full peer review and any attached files.

If you choose “no”, your identity will remain anonymous but your review may still be made public.

**Do you want your identity to be public for this peer review?** For information about this choice, including consent withdrawal, please see our Privacy Policy.

Reviewer #1: No

[NOTE: If reviewer comments were submitted as an attachment file, they will be attached to this email and accessible via the submission site. Please log into your account, locate the manuscript record, and check for the action link "View Attachments". If this link does not appear, there are no attachment files.]

While revising your submission, please upload your figure files to the Preflight Analysis and Conversion Engine (PACE) digital diagnostic tool, https://pacev2.apexcovantage.com/. PACE helps ensure that figures meet PLOS requirements. To use PACE, you must first register as a user. Registration is free. Then, login and navigate to the UPLOAD tab, where you will find detailed instructions on how to use the tool. If you encounter any issues or have any questions when using PACE, please email PLOS at figures@plos.org. Please note that Supporting Information files do not need this step.

# Author comment: Author response to Decision Letter 0

**DOI:** 10.1371/journal.pone.0261963.r002

4 Nov 2021

Dear Dr. Magar,

Thank you very much for the helpful comments on our manuscript titled “Great egret (Ardea alba) habitat selection and foraging behavior in a temperate estuary: comparing natural wetlands to areas with shellfish aquaculture”. We appreciate the feedback from you and the reviewer and feel that collectively it made our paper stronger. Attached please find a marked up and unmarked versions of our paper, all figure and supporting information files, and a text version of an email correspondence regarding rights to use map material. 

We have addressed all comments and suggestions by you and the reviewer (see red below). In nearly every instance we made the recommended change but see below for justification where we did not make the recommended change. The most substantial change we made is the inclusion of 3 additional models in our candidate set for evaluating habitat selection. The original best-supported model is still the best supported and remains the only model we base inference on for that part of the paper. However, we appreciate the reviewer’s comments and feel that the larger candidate set provides a fuller evaluation of the patterns we observed.

Thank you very much for your time and for considering our manuscript for publication.

Scott Jennings

Journal Requirements:

When submitting your revision, we need you to address these additional requirements.

1. Please ensure that your manuscript meets PLOS ONE's style requirements, including those for file naming. The PLOS ONE style templates can be found at 

https://journals.plos.org/plosone/s/file?id=wjVg/PLOSOne_formatting_sample_main_body.pdf and 

https://journals.plos.org/plosone/s/file?id=ba62/PLOSOne_formatting_sample_title_authors_affiliations.pdf

We have reviewed our submission against the style requirements. To the best of our knowledge our submission meets all requirements.

2. Please review your reference list to ensure that it is complete and correct. If you have cited papers that have been retracted, please include the rationale for doing so in the manuscript text, or remove these references and replace them with relevant current references. Any changes to the reference list should be mentioned in the rebuttal letter that accompanies your revised manuscript. If you need to cite a retracted article, indicate the article’s retracted status in the References list and also include a citation and full reference for the retraction notice.

We have reviewed our reference list. We note that in the original submission we cited unpublished data from the California Department of Fish and Wildlife as (CDFW, unpublished data). We have replaced this text with an inline citation number following instructions for citations, and in our reference list for the appropriate inline number we have added “California Department of Fish and Wildlife. Tomales Bay shellfish production, unpublished data.”

3. In your Methods section, please provide additional location information of the study site, including geographic coordinates for the data set if available. We have added coordinates

4. We note that the grant information you provided in the ‘Funding Information’ and ‘Financial Disclosure’ sections do not match. 

When you resubmit, please ensure that you provide the correct grant numbers for the awards you received for your study in the ‘Funding Information’ section. We received no grants to conduct this work.

5. Thank you for stating the following in the Acknowledgments Section of your manuscript: 

"Funding came from generous individual donors to Audubon Canyon Ranch and through the continued support of its Board of Directors. None of our funders had any influence on the content of the manuscript."

We note that you have provided funding information that is not currently declared in your Funding Statement. However, funding information should not appear in the Acknowledgments section or other areas of your manuscript. We will only publish funding information present in the Funding Statement section of the online submission form. We have deleted funding text from our Acknowledgements section.

Please remove any funding-related text from the manuscript and let us know how you would like to update your Funding Statement. Currently, your Funding Statement reads as follows: 

"Funding came from generous individual donors to Audubon Canyon Ranch and through the continued support of its Board of Directors. None of our funders had any influence on the content of the manuscript."

Please include your amended statements within your cover letter; we will change the online submission form on your behalf.

Please keep our funding statement as it currently reads.

6. Your ethics statement should only appear in the Methods section of your manuscript. If your ethics statement is written in any section besides the Methods, please move it to the Methods section and delete it from any other section. Please ensure that your ethics statement is included in your manuscript, as the ethics statement entered into the online submission form will not be published alongside your manuscript. We have moved our ethics statement to Methods, in the first paragraph of the trapping/tagging subsection.

7. We note that Figure(s) 1 in your submission contain [map/satellite] images which may be copyrighted. All PLOS content is published under the Creative Commons Attribution License (CC BY 4.0), which means that the manuscript, images, and Supporting Information files will be freely available online, and any third party is permitted to access, download, copy, distribute, and use these materials in any way, even commercially, with proper attribution. For these reasons, we cannot publish previously copyrighted maps or satellite images created using proprietary data, such as Google software (Google Maps, Street View, and Earth). For more information, see our copyright guidelines: http://journals.plos.org/plosone/s/licenses-and-copyright.

We have contacted ESRI regarding copyright status of material included in our figure 1. According to the email correspondence copied to the end of this document as well as the following webpage (https://doc.arcgis.com/en/arcgis-online/reference/static-maps.htm), we feel that we have sufficient permission to use these materials. We have added attribution for these materials to our figure caption.

We require you to either (1) present written permission from the copyright holder to publish these figures specifically under the CC BY 4.0 license, or (2) remove the figures from your submission:

a. You may seek permission from the original copyright holder of Figure(s) 1 to publish the content specifically under the CC BY 4.0 license. 

We recommend that you contact the original copyright holder with the Content Permission Form (http://journals.plos.org/plosone/s/file?id=7c09/content-permission-form.pdf) and the following text:

“I request permission for the open-access journal PLOS ONE to publish XXX under the Creative Commons Attribution License (CCAL) CC BY 4.0 (http://creativecommons.org/licenses/by/4.0/). Please be aware that this license allows unrestricted use and distribution, even commercially, by third parties. Please reply and provide explicit written permission to publish XXX under a CC BY license and complete the attached form.”

Please upload the completed Content Permission Form or other proof of granted permissions as an "Other" file with your submission.

In the figure caption of the copyrighted figure, please include the following text: “Reprinted from [ref] under a CC BY license, with permission from [name of publisher], original copyright [original copyright year].”

b. If you are unable to obtain permission from the original copyright holder to publish these figures under the CC BY 4.0 license or if the copyright holder’s requirements are incompatible with the CC BY 4.0 license, please either i) remove the figure or ii) supply a replacement figure that complies with the CC BY 4.0 license. Please check copyright information on all replacement figures and update the figure caption with source information. If applicable, please specify in the figure caption text when a figure is similar but not identical to the original image and is therefore for illustrative purposes only.

The following resources for replacing copyrighted map figures may be helpful:

USGS National Map Viewer (public domain): http://viewer.nationalmap.gov/viewer/

The Gateway to Astronaut Photography of Earth (public domain): http://eol.jsc.nasa.gov/sseop/clickmap/

Maps at the CIA (public domain): https://www.cia.gov/library/publications/the-world-factbook/index.html and https://www.cia.gov/library/publications/cia-maps-publications/index.html

NASA Earth Observatory (public domain): http://earthobservatory.nasa.gov/

Landsat: http://landsat.visibleearth.nasa.gov/

USGS EROS (Earth Resources Observatory and Science (EROS) Center) (public domain): http://eros.usgs.gov/#

Natural Earth (public domain): http://www.naturalearthdata.com/

[Note: HTML markup is below. Please do not edit.]

Reviewers' comments:

Reviewer's Responses to Questions

Comments to the Author

1. Is the manuscript technically sound, and do the data support the conclusions?

The manuscript must describe a technically sound piece of scientific research with data that supports the conclusions. Experiments must have been conducted rigorously, with appropriate controls, replication, and sample sizes. The conclusions must be drawn appropriately based on the data presented. 

Reviewer #1: Yes

________________________________________

2. Has the statistical analysis been performed appropriately and rigorously? 

Reviewer #1: Yes

________________________________________

3. Have the authors made all data underlying the findings in their manuscript fully available?

The PLOS Data policy requires authors to make all data underlying the findings described in their manuscript fully available without restriction, with rare exception (please refer to the Data Availability Statement in the manuscript PDF file). The data should be provided as part of the manuscript or its supporting information, or deposited to a public repository. For example, in addition to summary statistics, the data points behind means, medians and variance measures should be available. If there are restrictions on publicly sharing data—e.g. participant privacy or use of data from a third party—those must be specified.

Reviewer #1: Yes

________________________________________

4. Is the manuscript presented in an intelligible fashion and written in standard English?

PLOS ONE does not copyedit accepted manuscripts, so the language in submitted articles must be clear, correct, and unambiguous. Any typographical or grammatical errors should be corrected at revision, so please note any specific errors here.

Reviewer #1: Yes

________________________________________

5. Review Comments to the Author

Please use the space provided to explain your answers to the questions above. You may also include additional comments for the author, including concerns about dual publication, research ethics, or publication ethics. (Please upload your review as an attachment if it exceeds 20,000 characters)

Reviewer #1: Line 22 insert comma after marsh added

Line 79 need space between [16] and comma It is unclear whether the reviewer is referring to the comma inside the brackets or outside. If outside, we believe there shouldn’t be a space before the comma. If inside, the Plos One style template linked above indicates there should be no spaces separating multiple citations inside brackets.

Line 116 labeled with we have changed the wording of this sentence to be more clear.

Line 117 eelgrass shown in dark gray and , shellfish…. We disagree that a comma should be added here. We have eliminated the semi-colon to make this two sentences for ease of reading.

Line 137 Does Publicly have to be capitalized. Hyphen is not necessary hyphen removed; we do not believe Publicly should be capitalized.

Line 138 end sentence after ...our analysis. Done

Line 139 Capital W to start line Done

Line 151 remove a after in GIS…. We disagree that this “a” should be removed. We are referring here to a single object.

Line 182 I did not find that DEM was defined prior to this sentence, will your reader know what this is? We have added “(DEM)” at line 165 where this term is first used.

Line 185 end sentence after collected Done

Line 186 start line with capital T Done

Line 198 add space between ) and [5] Done

Line 206 delete dur to Done

Line 228 “third order selection” needs to be better defined if the concept of Johnson’s hierarchical habitat selection is used. This term has not been described either in the introduction or methods prior to this point. We have added some text in parentheses to this sentence clarifying the distinction between first, second and third order resource selection. We feel that the concept of hierarchical resource selection is well enough understood and accepted in ecological research (the Johnson 1980 paper we cite has >3950 citations according to Google scholar) that further explanation is not warranted here. If the editor disagrees with this conclusion, we can add more text, but in the interest of maintaining overall readability of this paper we feel the current text is sufficient. Note also that we have moved this sentence to the start of the following paragraph where we feel it fits better.

L.232-233 “evaluate fine scale habitat selection but avoid an excessive number of steps with no movement” – this data thinning may result in bias: “stay-put” during foraging may suggest selection, and more residence time. You may need movement steps for non-zero step selection functions; however, the more the locations you filtered out, the more the information of preference (staying there longer) you may lose. Caution needs to be exercised here. This is a fair point; however, it should be noted that any sampling frequency that researchers select may yield different results than other possible sampling frequencies. Our filtering was done to avoid GPS measurement error being represented in our step length values, though we appreciate that this needed to be better explained. As demonstrated by Ranacher (2016), GPS measurement error can lead to systematic overestimation of movement distances when sampling intervals are short relative to movement speeds. This can be particularly problematic as sampling interval decreases to a degree that the step length over that interval approaches the GPS tag measurement error (Noonan 2019). Our preliminary data summarization revealed that median step lengths at the 5 minute sampling interval were of similar magnitude as the tag-estimated measurement error (3-10m; see figure below), whereas at the 10 minute interval median step lengths were ≥3-4 times longer than tag error. Thus, our use of the 10 minute interval represents the shortest interval that would yield reliable estimates of movement distances, given the inherent measurement error of the GPS tags we used. We have expanded the description of our “preliminary data summarization” and subsequent rationale for choosing 10 minutes here.

Noonan MJ, Fleming CH, Akre TS, Drescher-Lehman J, Gurarie E, Harrison AL, et al. Scale-insensitive estimation of speed and distance traveled from animal tracking data. Mov Ecol. 2019;7: 1–15. doi:10.1186/s40462-019-0177-1

Ranacher P, Brunauer R, Trutschnig W, Der S Van, Reich S, Ranacher P, et al. Why GPS makes distances bigger than they are. Int J Geogr Inf Sci. 2016;30: 316–333. doi:10.1080/13658816.2015.1086924

Line 234 add space between ) and [33] Done

Line 245 What were an example of the observations of foraging behavior that prompted you to select this scale, an explanation would help the reader. We have deleted the text referring to foraging behavior and habitat structure from this sentence. We believe that basing our habitat raster resolution on the accuracy of our GPS tags is sufficient justification.

Line 248 what is a small number, state how many models, be specific Done

L. 252-253 The time-interval that you “chose” may affect the model outcomes because birds may choose to stay longer with shorter step length if wetland types are desirable; however, a part of this information has been thrown out by filtering the data. A sensitivity analysis as the authors mentioned (L. 246-247) is critical. See our response to the reviewer’s similar comment at line 232-233. We believe that the suggested sensitivity analysis is not required, but if the editor feels it is necessary we can conduct it.

Line 252-254 you chose to evaluate wetland type at the start or end of the step. Use this same order in the next sentence We have restructured this paragraph slightly to be more clear about which models were used for which part of our analysis. This suggestion is no longer relevant.

Line 256 than for natural wetlands added “for”

Line 257 “interactions between the 4-level wetland type variable and time-specific water depth”: have you checked whether the wetland types and water depth are correlated before they are included in the same model? From a strictly statistical perspective, one cannot calculate a Pearson correlation between continuous and categorical variables. However, the reviewer makes a good point here and on their comment for lines 320-332. Thus, we have added a plot as supplementary material showing the distribution of elevations for each wetland type. We feel that the ranges of elevations for each wetland type in our original analysis overlap sufficiently for our comparison to be valid. Nevertheless, because shellfish aquaculture wetland type is of primary interest in our analysis and because there were relatively few shellfish aquaculture raster cells with depths < -0.5 m (i.e., 0.5 m above the tidal level), we also restricted our analysis of habitat selection to depths ≥ -0.5 m to minimize any effect of this data sparseness on our results. This data filtering added some nuance to the comparison of selection among natural wetlands but does not change the observed patterns or ecological conclusions about shellfish aquaculture. We have edited the text here in Methods, as well as in Results and Discussion to reflect this change.

Line 261 … we deemed a quadratic response to be the most biologically likely… based on what? How did you determine this approach was the most biologically likely? These are fair questions. We mostly based this on our observations of egrets foraging in the study system through the tidal cycle, and what is generally known about Great Egret foraging water depth preferences. However, we appreciate that this rationale may not satisfy the editor or readers, and in the interest of conducting a more complete investigation we have added 3 models to the original candidate set of 4 models. These new models investigate linear effects of water depth: wetland type * depth; wetland type + depth; depth. As can be seen in our new S1 Table, these linear depth models were very poorly supported by the data, with Delta AICc values between 295-1766. Thus, while we now provide model selection results for the full 7 model candidate set, our results and inference are based on the same models as in the original submission. We have edited the text in this section of the manuscript to reflect this new candidate model set.

Line 261-262 The authors decided to use quadratic functions as described in the methods section without further justification using the literature or some preliminary analysis. The relationship with other three wetland types appeared to be linear based on Fig. 2. See comment above.

Line 264 add space between ) and [37] Done

Line 301 Table 1 do you have an explanation as to the egrets that quit (?) sending data in 2018 and 2019? Did they leave the area due to migration? If so, did they revisit the area? Did you have any transmitter failure? Did you have mortality? We have added 2 short sentences to the first paragraph of Results providing this information.

Lines 320-332 To better support and clarify the statements, a box plot of tidal water depth for four wetland types (maybe as a supplementary figure) would be useful. This is a good suggestion, we have added a plot to show the distribution of elevations for each wetland type as a supplementary figure.

Line 331-332 … natural wetlands than for shellfish aquaculture areas across most water depths… define most, number, percentage? Hard to determine how important this is. We have deleted this sentence, as it repeats what was written earlier in the paragraph. We believe that the text earlier in this paragraph provides sufficient detail to understand our results.

Line 372 delete they Done

Line 393 aquaculture areas were well within … We disagree with this word change (“was” to “were”). The noun in this sentence is the collective ability of all tagged egrets to fly a certain distance, which is singular. Thus this sentence employs third person past tense, singular, in which case “was” is the correct form of “to be”.

Lines 398-399 “…showed a strong quadratic response across the range of water depths we investigated,…” Does this suggest your model only fit data on shellfish aquaculture well, failing on the other three wetland types? No. A quadratic predictor can fit the response variable just as well as a linear predictor (by fitting a very small value to the squared term); in such cases the quadratic predictor won’t “fail”, it will simply be penalized in AIC model selection for having an extra parameter. Because we included the interaction term between water depth and wetland type in our models, we allowed the form of the quadratic water depth response to be fit separately to each wetland type. This interaction term allowed the maximum likelihood procedure to find coefficient values that optimized the fitted values for each wetland types separately, rather than finding a single coefficient value that adequately fit all wetland types. The reviewer’s concern would be correct had we only fit the model with the additive wetland type and water depth terms (“wetland type + depth2” in S1 Table). Indeed, the very large Delta AICc values for this model among all birds (S1 Table) provide strong evidence that resource selection takes a substantially different form in each wetland type. We have added text to the third paragraph of the Results (lines 316-317) clarifying this. See also our reply to the reviewer’s comment for line 261.

Line 459 insert comma after marsh Done

Overview:

This is an important study because there has been little attention to the relationships between shellfish aquaculture and wading-bird habitat selection during the past. An interesting part of this study is the quantification of foraging activities with movement speed and activity level (with accelerometer data). Their results of the analyses look fine from their reports of AICc values and Figures. However, the quadratic curve is only obvious with shellfish aquaculture in Figure 2. As the tidal water depth increased between 0 and 1, relative selection strength converged among the four types of wetlands (Fig. 2). The authors decided to use quadratic functions as described in the method section without further justification using the literature or some preliminary analysis (L.261-262). The relationship with other three wetland types appeared to be linear based on Fig. 2. To better support and clarify the statements between lines 320-332, a box plot of tidal water depth for the four wetland types (maybe as a supplementary figure) would be useful. See our replies to specific lines above.

The actual description of their statistical analysis is good, but my main concerns are twofold. 1) sample size of seven birds is too small for a reliable inference even when analysis is by individual bird; and 2) data filtering may have affected the results and conclusions. We have added cautionary text to the first paragraph of our Discussion, noting the small sample size and the descriptive rather than predictive nature of our results. We have also further justified our data filtering to describe how we used the finest temporal scale that was possible given the inherent error of our GPS tags.

Email correspondence with ESRI regarding map copyrights:

From: Emiko Condeso

Sent: Thursday, August 12, 2021 9:05 PM

To: Scott Jennings

Subject: FW: Esri Case #02864788 - Proper attribution for a map made with data from the Living 

Atlas

Hi Scott,

See below for the answer from ESRI regarding publication permission for their data layers. I actually 

think this link might more explicitly show that ESRI permits this use: 

https://doc.arcgis.com/en/arcgis-online/reference/static-maps.htmI

The attribution needed for the ESRI terrain layer is:

Source: Airbus, USGS, NGA, NASA, CGIAR, NLS, OS, NMA, Geodatastyrelsen, GSA, GSI 

and the GIS User Community

Attribution for the state boundary layer is:

Sources: Esri, TomTom, U.S. Department of Commerce, U.S. Census Bureau

Also included in the map that would need attribution are:

Eelgrass: SFEI (I emailed SFEI to check for proper attribution on maps, in case they want us to cite the 

local Tomales mappers rather than them, but no answer), county boundary layer: California Department 

of Forestry and Fire Protection, shellfish gear: ACR.

Cheers,

Emi

T. Emiko Condeso 

Ecologist/GIS Specialist, Cypress Grove Research Center

My pandemic work week is M-F, 3-5pm. 

Due to my family/work balance you might receive messages 

from me outside normal working hours. Please do not feel 

pressured to reply outside your usual work schedule.

Audubon Canyon Ranch

P.O. Box 808, Marshall, CA 94940

P: 415-663-8203 ext. 401 | C: 707-364-3274 

emiko.condeso@egret.org | WEB: egret.org

Connecting nature, people and science in a rapidly changing world

From: Esri Customer Care <customercare@esri.com> 

Sent: Monday, August 9, 2021 2:10 PM 

To: Emiko Condeso <emiko.condeso@egret.org> 

Subject: Esri Case #02864788 - Proper attribution for a map made with data from the Living Atlas

Hello Emiko, 

Thank you for replying to my email. To answer your question, yes they are (provided that proper credits 

as listed in the Item Details page are given). 

I hope this helps! Other than that, the link below should provide further clarification: 

* Terms of use for items: https://doc.arcgis.com/en/arcgis-online/reference/access-use-

constraints.htm

Please feel free to let me know if you have any additional questions. Looking forward to hearing from 

you soon! 

Regards, 

Dalilah 

Esri Support Services 

https://my.esri.com/#/support/cases/02864788

https://support.esri.com/en/

THE SCIENCE OF WHERE™ 

ref:_00D70JXts._5005x1dXQYm:ref 

--------------- Original Message --------------- 

From: Emiko Condeso [emiko.condeso@egret.org] 

Sent: 07/08/2021 10:56 

To: 

Subject: RE: Esri Case #02864788 - Proper attribution for a map made with data from the Living Atlas 

Original Recipients: 

To: Esri Customer Care <customercare@esri.com>,

Cc: []

Thank you for this information. Are you telling me that the data are free to publish under a Creative 

Commons Attribution License, as long as I use the appropriate attribution? I need written permission 

for the publisher.

Thank you,

Emiko

T. Emiko Condeso 

Ecologist/GIS Specialist, Cypress Grove 

Research Center

My pandemic work week is M-F, 3-5pm. 

Due to my family/work balance you 

might receive messages from me outside 

normal working hours. Please do not feel 

pressured to reply outside your usual 

work schedule.

image001.jpg

[egret.org] 

Audubon Canyon Ranch

P.O. Box 808, Marshall, CA 94940

P: 415-663-8203 ext. 401 | C: 707-364-

3274 

emiko.condeso@egret.org | WEB: 

egret.org [egret.org] 

 image002.png

[facebook.com] 

 image003.png

[instagram.com] 

 image004.png

[twitter.com] 

 image005.png

[vimeo.com] 

Connecting nature, people and science in a rapidly changing world

image006.png

From: Esri Customer Care <customercare@esri.com> 

Sent: Friday, August 6, 2021 8:22 AM 

To: Emiko Condeso <emiko.condeso@egret.org> 

Subject: Esri Case #02864788 - Proper attribution for a map made with data from the Living Atlas

Hello Emiko, 

Thank you for being patient while we are looking into this query. For your information, the Credits 

(Attribution) section in the data's Item Details page is where users can find the appropriate attribution 

for credits and appreciation section if they are using the data in their journals. Users should always 

credit the sources found in the Credits field of the item page for an ArcGIS Online map. Example below is 

the Credits (attribution) section for the multi-directional hillshade data: 

ima

ge0

07.p

ng

You may navigate to this link to view the Item Details page of the data and scroll down on the page until 

you find a Credits (Attribution) section. Since the section already listed several organizations that should 

be credited, you may use the list in the section to give credits to the organizations. 

I hope this helps! Please let me know if you have any additional questions. Looking forward to hearing 

from you soon. 

Thank you and keep safe. 

Regards, 

Dalilah 

Esri Support Services 

https://my.esri.com/#/support/cases/02864788

https://support.esri.com/en/

THE SCIENCE OF WHERE™ 

ref:_00D70JXts._5005x1dXQYm:ref 

--------------- Original Message --------------- 

From: Emiko Condeso [emiko.condeso@egret.org] 

Sent: 06/08/2021 08:56 

To: 

Subject: RE: Esri Case #02864788 - Proper attribution for a map made with data from the Living Atlas 

Original Recipients: 

To: Esri Customer Care <customercare@esri.com>,

Cc: []

Thank you!

T. Emiko Condeso 

Ecologist/GIS Specialist, Cypress Grove Research Center

My pandemic work week is M-F, 3-5pm. 

Due to my family/work balance you might receive 

messages from me outside normal working hours. Please do 

not feel pressured to reply outside your usual work 

schedule.

ima

ge0

01.j

pg

[egret.org]

Audubon Canyon Ranch

P.O. Box 808, Marshall, CA 94940

P: 415-663-8203 ext. 401 | C: 707-364-3274 

emiko.condeso@egret.org | WEB: egret.org

[egret.org] 

ima

ge0

02.p

ng

[facebook.com] 

ima

ge0

03.p

ng

[instagram.com] 

ima

ge0

04.p

ng

[twitter.com] 

ima

ge0

05.p

ng

[vimeo.com] 

Connecting nature, people and science in a rapidly changing world

ima

ge0

06.p

ng

From: Esri Customer Care <customercare@esri.com> 

Sent: Thursday, August 5, 2021 4:42 PM 

To: Emiko Condeso <emiko.condeso@egret.org> 

Subject: Esri Case #02864788 - Proper attribution for a map made with data from the Living Atlas

Hello Emiko, 

Warm greetings and good day to you. 

This is Dalilah from ESRI Support Services, writing in reference to the case #02864788. I have taken 

ownership of the case and will be working with you to reach a resolution. 

As per notification received, I understand that you have some queries about proper attribution for a 

map made with data from the Living Atlas, mainly whether these data are available to freely publish 

with proper attribution. I also understand that the map will be published by a journal under Creative 

Commons Attribution License, which requires written permission by the copywrite holder for 

publication. 

I would appreciate if you could allow me some time to perform adequate research and to consult this 

internally with my team before getting back to you with more information. I appreciate your patience 

while this process takes place. 

In the meantime, have a good rest of your day and keep safe! 

Regards, 

Dalilah 

Esri Support Services 

https://my.esri.com/#/support/cases/02864788

https://support.esri.com/en/

THE SCIENCE OF WHERE™ 

ref:_00D70JXts._5005x1dXQYm:ref 

Learn more about the Esri Support [play.google.com] [itunes.apple.com] 

Learn more about the Esri Support [play.google.com] [itunes.apple.com] 

Learn more about the Esri Support

Attachment Submitted filename: Response to Reviewers.docx Click here for additional data file.

# Peer review: Decision Letter 1

**DOI:** 10.1371/journal.pone.0261963.r003

**Authors:** Hans-Uwe Dahms

15 Dec 2021

Great egret (Ardea alba) habitat selection and foraging behavior in a temperate estuary: comparing natural wetlands to areas with shellfish aquaculture

PONE-D-21-19522R1

Dear Dr. JENNINGS,

We’re pleased to inform you that your manuscript has been judged scientifically suitable for publication and will be formally accepted for publication once it meets all outstanding technical requirements.

Within one week, you’ll receive an e-mail detailing the required amendments. When these have been addressed, you’ll receive a formal acceptance letter and your manuscript will be scheduled for publication.

An invoice for payment will follow shortly after the formal acceptance. To ensure an efficient process, please log into Editorial Manager at http://www.editorialmanager.com/pone/, click the 'Update My Information' link at the top of the page, and double check that your user information is up-to-date. If you have any billing related questions, please contact our Author Billing department directly at authorbilling@plos.org.

If your institution or institutions have a press office, please notify them about your upcoming paper to help maximize its impact. If they’ll be preparing press materials, please inform our press team as soon as possible -- no later than 48 hours after receiving the formal acceptance. Your manuscript will remain under strict press embargo until 2 pm Eastern Time on the date of publication. For more information, please contact onepress@plos.org.

Kind regards,

Hans-Uwe Dahms, Ph.D.

Academic Editor

PLOS ONE

Additional Editor Comments (optional):

ACCEPTED

Reviewers' comments:

Reviewer's Responses to Questions

**Comments to the Author**

1. If the authors have adequately addressed your comments raised in a previous round of review and you feel that this manuscript is now acceptable for publication, you may indicate that here to bypass the “Comments to the Author” section, enter your conflict of interest statement in the “Confidential to Editor” section, and submit your "Accept" recommendation.

Reviewer #2: (No Response)

**********

2. Is the manuscript technically sound, and do the data support the conclusions?

The manuscript must describe a technically sound piece of scientific research with data that supports the conclusions. Experiments must have been conducted rigorously, with appropriate controls, replication, and sample sizes. The conclusions must be drawn appropriately based on the data presented. 

Reviewer #2: Yes

**********

3. Has the statistical analysis been performed appropriately and rigorously? 

Reviewer #2: Yes

**********

4. Have the authors made all data underlying the findings in their manuscript fully available?

The PLOS Data policy requires authors to make all data underlying the findings described in their manuscript fully available without restriction, with rare exception (please refer to the Data Availability Statement in the manuscript PDF file). The data should be provided as part of the manuscript or its supporting information, or deposited to a public repository. For example, in addition to summary statistics, the data points behind means, medians and variance measures should be available. If there are restrictions on publicly sharing data—e.g. participant privacy or use of data from a third party—those must be specified.

Reviewer #2: Yes

**********

5. Is the manuscript presented in an intelligible fashion and written in standard English?

PLOS ONE does not copyedit accepted manuscripts, so the language in submitted articles must be clear, correct, and unambiguous. Any typographical or grammatical errors should be corrected at revision, so please note any specific errors here.

Reviewer #2: No

**********

6. Review Comments to the Author

Please use the space provided to explain your answers to the questions above. You may also include additional comments for the author, including concerns about dual publication, research ethics, or publication ethics. (Please upload your review as an attachment if it exceeds 20,000 characters)

Reviewer #2: Main comments:

1. The reason why the shellfish aquaculture was only selected at a narrow range of water depths probably because it is not the prime foraging habitat for great egrets. During low tide period, great egrets have mass natural habitats to forage; therefore, the relative selection strength for shellfish aquaculture decreased rapidly. In addition, after shellfish aquaculture infrastructures are exposed above water level, it would be difficult for great egrets to forage around. However, when tide comes up, the shellfish aquaculture is the last area to be inundated and thus becomes a potential foraging site for great egrets. During high tide, shellfish aquaculture was used more frequently probably because of its location, with higher elevation. If the proposition is right, the most important factor affecting the foraging of the great egrets would be the availability of natural wetlands. They would always prefer natural habitats unless high tide pushes them away.

2. Since the GPS-datalogger cannot provide information on foraging success, it is no way to tell a great egret is foraging or resting in a particular habitat. Especially, “sit and wait” is one of the foraging tactics they apply frequently. As a result, movement speed might not directly reflect foraging success.

3. Is it OK to turn the graph around along the X axis in Figure 2? Making negative values for data below water level would be easily to understand.

4. Based on Fig. 3 and 4, it appears that great egrets used more short movements and spent less energy (ODBA) in tidal marsh than in other habitat types. I do not know if it implies that great egrets forage more frequently in tidal marsh.

5. In Figure 4, it is better to designate the difference among wetland types. For example, mark different letters (a, b, or c) to show significant difference based on the Tukey’s test or non-parametric analysis.

6. In discussion, some results could be explained more explicitly. For example, how tidal movement affects the foraging behavior of great egrets and why shellfish aquaculture was selected at a narrow range of water depth? In addition, the disturbance of human activity in shellfish aquaculture was not discussed at all. It could be an important factor preventing great egrets to use shellfish aquaculture.

Others:

L25-26: “We found evidence for stronger overall habitat selection for natural wetlands than for shellfish aquaculture areas,…”

When we refer to habitat selection, the availability of each habitat type must be taken into consideration. A use and availability analysis is usually required before any conclusion on habitat selection is made; otherwise the result is about habitat use, not habitat selection. Although the authors applied iSSA (integrated step selection analysis), the results might not agree with a use and availability analysis.

L206: due to not dur to.

L296: ”We obtained a mean = 177 ± SE 31 days of Tomales Bay foraging data per tagged great egret,…”

Actually, we do not know if great egrets are really foraging based on GPS data even though data have been filtered before data analysis.

In Figures 2 and 3, GREG_10 was located between GREG_1 and GREG_2. It is better to arrange them in order. The authors probably can rename the first 6 by GREG_01, GREG_02, etc, and the software will place the GREG_10 into the last place.

**********

7. PLOS authors have the option to publish the peer review history of their article (what does this mean?). If published, this will include your full peer review and any attached files.

If you choose “no”, your identity will remain anonymous but your review may still be made public.

**Do you want your identity to be public for this peer review?** For information about this choice, including consent withdrawal, please see our Privacy Policy.

Reviewer #2: No

# Editor report: Acceptance letter

**DOI:** 10.1371/journal.pone.0261963.r004

**Authors:** Hans-Uwe Dahms

21 Dec 2021

PONE-D-21-19522R1 

Great egret (*Ardea alba*) habitat selection and foraging behavior in a temperate estuary: comparing natural wetlands to areas with shellfish aquaculture 

Dear Dr. Jennings:

I'm pleased to inform you that your manuscript has been deemed suitable for publication in PLOS ONE. Congratulations! Your manuscript is now with our production department. 

If your institution or institutions have a press office, please let them know about your upcoming paper now to help maximize its impact. If they'll be preparing press materials, please inform our press team within the next 48 hours. Your manuscript will remain under strict press embargo until 2 pm Eastern Time on the date of publication. For more information please contact onepress@plos.org.

If we can help with anything else, please email us at plosone@plos.org. 

Thank you for submitting your work to PLOS ONE and supporting open access. 

Kind regards, 

PLOS ONE Editorial Office Staff

on behalf of

Dr. Hans-Uwe Dahms 

Academic Editor

PLOS ONE
